# *Zeb1* modulates hematopoietic stem cell fates required for suppressing acute myeloid leukemia

**DOI:** 10.1172/JCI129115

**Published:** 2021-01-04

**Authors:** Alhomidi Almotiri, Hamed Alzahrani, Juan Bautista Menendez-Gonzalez, Ali Abdelfattah, Badi Alotaibi, Lubaid Saleh, Adelle Greene, Mia Georgiou, Alex Gibbs, Amani Alsayari, Sarab Taha, Leigh-anne Thomas, Dhruv Shah, Sarah Edkins, Peter Giles, Marc P. Stemmler, Simone Brabletz, Thomas Brabletz, Ashleigh S. Boyd, Florian A. Siebzehnrubl, Neil P. Rodrigues

**Affiliations:** 1 European Cancer Stem Cell Research Institute, Cardiff University, School of Biosciences, Cardiff, United Kingdom.; 2 College of Applied Medical Sciences-Dawadmi, Shaqra University, Dawadmi, Saudi Arabia.; 3 Wales Gene Park and Wales Cancer Research Centre, Division of Cancer and Genetics, Cardiff University, School of Medicine, Cardiff, United Kingdom.; 4 Department of Experimental Medicine 1, Nikolaus-Fiebiger-Center for Molecular Medicine, FAU University Erlangen-Nürnberg, Erlangen, Germany.; 5 Department of Surgical Biotechnology, Division of Surgery and Interventional Science, Royal Free Hospital, and; 6 Institute of Immunity and Transplantation, University College London, London, United Kingdom.

**Keywords:** Hematology, Stem cells, Bone marrow differentiation

## Abstract

*Zeb1*, a zinc finger E-box binding homeobox epithelial-mesenchymal transition (EMT) transcription factor, confers properties of “stemness,” such as self-renewal, in cancer. Yet little is known about the function of *Zeb1* in adult stem cells. Here, we used the hematopoietic system as a well-established paradigm of stem cell biology to evaluate *Zeb1*-mediated regulation of adult stem cells. We employed a conditional genetic approach using the *Mx1-Cre* system to specifically knock out (KO) *Zeb1* in adult hematopoietic stem cells (HSCs) and their downstream progeny. Acute genetic deletion of *Zeb1* led to rapid-onset thymic atrophy and apoptosis-driven loss of thymocytes and T cells. A profound cell-autonomous self-renewal defect and multilineage differentiation block were observed in *Zeb1*-KO HSCs. Loss of *Zeb1* in HSCs activated transcriptional programs of deregulated HSC maintenance and multilineage differentiation genes and of cell polarity consisting of cytoskeleton-, lipid metabolism/lipid membrane–, and cell adhesion–related genes. Notably, epithelial cell adhesion molecule (EpCAM) expression was prodigiously upregulated in *Zeb1*-KO HSCs, which correlated with enhanced cell survival, diminished mitochondrial metabolism, ribosome biogenesis, and differentiation capacity and an activated transcriptomic signature associated with acute myeloid leukemia (AML) signaling. *ZEB1* expression was downregulated in AML patients, and *Zeb1* KO in the malignant counterparts of HSCs — leukemic stem cells (LSCs) — accelerated *MLL-AF9–* and *Meis1a/Hoxa9*-driven AML progression, implicating *Zeb1* as a tumor suppressor in AML LSCs. Thus, *Zeb1* acts as a transcriptional regulator in hematopoiesis, critically coordinating HSC self-renewal, apoptotic, and multilineage differentiation fates required to suppress leukemic potential in AML.

## Graphical abstract

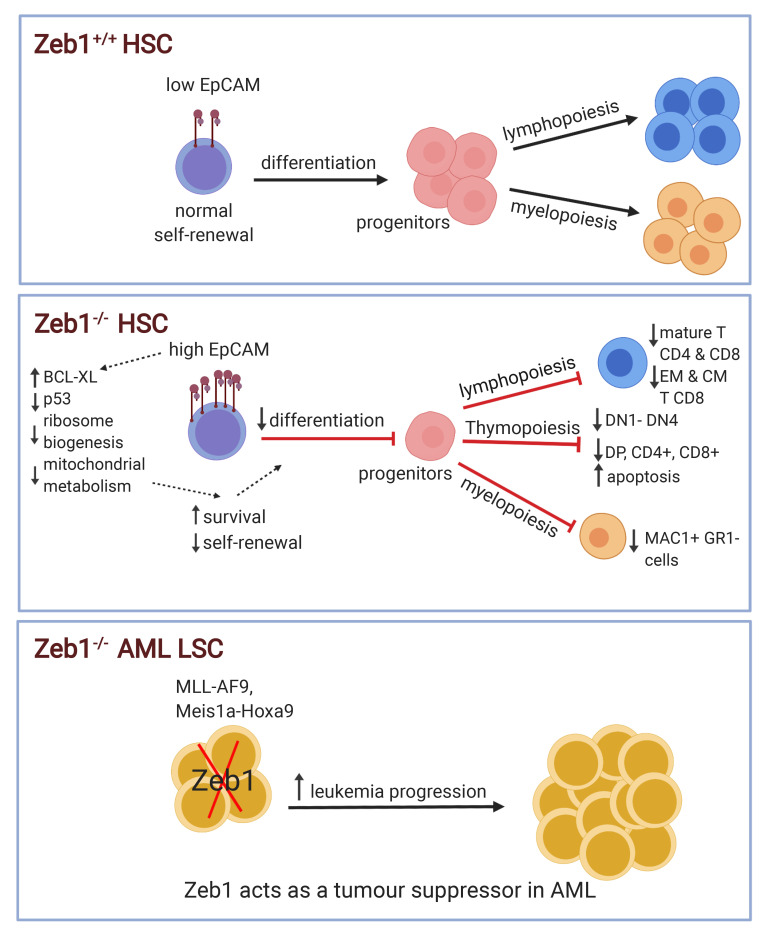

## Introduction

Epithelial-mesenchymal transition (EMT) is a complex process that organizes specific changes in cellular fate and phenotype and is usually accompanied by loss of cell polarity and adhesion and increased locomotion (1). EMT is an important step in embryonic development and regeneration, which largely promotes a program of cellular plasticity and migration (2). This program is regulated by specific transcription factors (TFs), such as members of the ZEB, SNAI, and TWIST families. To this end, *Zeb1*, a zinc finger TF that binds to E-box motifs, has been implicated in myogenesis (3–6), neuronal development and differentiation (7–10), postgastrulation embryogenesis (11) and T cell development (12, 13).

It has also been posited that EMT is a critical regulator in cancer pathogenesis and, in particular, cancer stem cell behavior (14–16), which facilitates cancer cells becoming more metastatic, with resultant tumor progression (17). Abundant evidence shows that *Zeb1* regulates stem cell properties in cancer, including self-renewal (18–20). While it is established that *Zeb1* regulates expression of multiple stem cell–associated TFs, including those with oncogenic potential, such as BMI1, KLF4, and SOX2 (19, 21), and that loss of *Zeb1* promotes cellular differentiation during development of the embryonic CNS (9) and skeletal muscle (22), the wider role of *Zeb1* in normal stem cell–fate decisions remains unclear.

By exploiting a mouse model engineered to contain conditional alleles of *Zeb1* and an inducible *Mx-1-Cre* (*Zeb1^fl/fl^;Mx1-Cre^+^*) (23), where *Zeb1* expression can be deleted in hematopoietic stem cells (HSCs) and their progeny by administering polyinosinic-polycytidylic acid (pIpC), we used the hematopoietic system, as an established stem cell model, to evaluate *Zeb1*-mediated regulation of somatic stem cells. Herein, we identify *Zeb1* as a crucial, indispensable regulator of adult T cell maturation and differentiation. In a broader context, as judged by conditional deletion within the hematopoietic system, we identify *Zeb1* as an essential transcriptional repressor balancing adult stem cell self-renewal, apoptotic, and global, multi-lineage differentiation fates of stem cells. Finally, we find that *Zeb1*-mediated regulation of these stem cell fates is required to suppress malignancy in the context of acute myeloid leukemia (AML).

## Results

### Acute conditional deletion of Zeb1 reduces the frequency of MAC1^+^ myeloid cells and CD8^+^ memory T cells.

**Figure 1:**
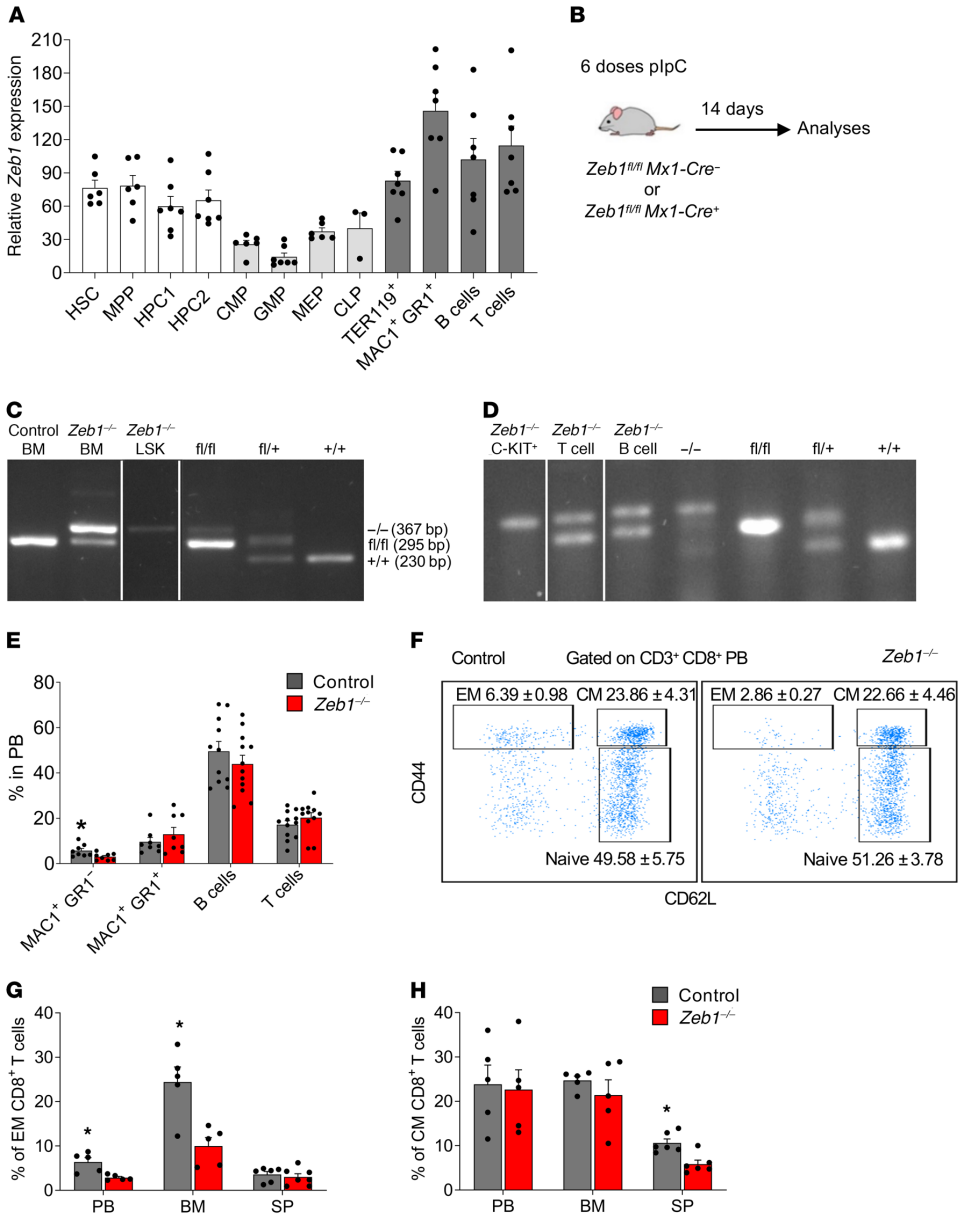
Loss of *Zeb1* affects effector and CM CD8^+^ T cells. (**A**) Q-PCR analysis of mRNA *Zeb1* expression in different hematopoietic populations (*n* = 6–7 except CLP *n* = 3). (**B**) Schematic of pIpC treatment to delete *Zeb1* in *Zeb1^fl/fl^;Mx1-Cre^–^* (control) and *Zeb1^fl/fl^;Mx1-Cre^+^* (*Zeb1^–/–^*) mice and analysis at day 14 after the last pIpC dose. (**C**) Representative gel electrophoresis analysis confirming *Zeb1* deletion in BM cells and LSK population 14 days after the last dose of pIpC. (**D**) Representative gel electrophoresis analysis of *Zeb1* deletion in BM C-KIT^+^ cells and spleen B (B220^+^) and T (CD3^+^) cells 14 days after the last dose of pIpC. (**E**) Frequency of differentiated cells in PB from control and *Zeb1^–/–^* mice 14 days after the last dose of pIpC from 4 independent experiments (*n* = 8–12 per group). (**F**) Gating strategy of naive, EM, and CM T cells using CD62L and CD44 markers along with T cell markers CD3, CD4, and CD8 in PB. Frequency of EM T cells (**G**) and CM T cells (**H**) within CD3^+^CD8^+^ T cells in PB, BM, and SP from control (*n* = 5 PB and BM, 6 SP) and *Zeb1^–/–^* (*n* = 5 PB and BM, 6–7 SP) mice from 2 independent experiments. Error bars show mean ± SEM. Mann-Whitney *U* test was used to calculate significance. *P < 0.05.

*Zeb1* expression has been observed in hematopoietic cells from BM, thymus, spleen, fetal liver, and lymph nodes (12, 24). However, the *Zeb1* expression pattern in different subsets of hematopoietic cells, including hematopoietic stem and progenitor cells (HSPCs), remains unclear. We therefore conducted quantitative PCR (Q-PCR) analysis of *Zeb1* expression in hematopoietic cell compartments prospectively isolated by FACS. *Zeb1* was expressed at high levels in stem and progenitor cells (HSCs, multipotent progenitor [MPP], hematopoietic progenitor cell 1 [HPC1], and HPC2) and in terminally differentiated cells (myeloid, erythroid, and B and T lineages) whereas it was lower in committed myeloid and lymphoid progenitors (common myeloid progenitors [CMP], granulocyte-monocyte progenitors [GMP], megakaryocyte-erythroid progenitors [MEP], common lymphoid progenitors [CLP]) (Figure 1A).

To evaluate the genetic requirement for *Zeb1* in adult HSCs, their progenitors, and fully differentiated blood and immune cells, we bred mice harboring conditional alleles of *Zeb1* (*Zeb1^fl/fl^* mice) (23) with *Mx1-Cre* (25) to obtain either *Zeb1^fl/fl^;Mx1-Cre^+/–^* or control (*Zeb1^fl/fl^;Mx1-Cre^–/–^*) mice and administered pIpC on alternate days for 10 days to achieve deletion of *Zeb1* (*Zeb1^–/–^*). Hematopoiesis in control or *Zeb1^–/–^* mice was analyzed 14 days after the last dose of pIpC (Figure 1B). *Zeb1* was partially deleted in total BM cells (Figure 1C). To assess whether *Zeb1* was completely deleted in HSCs from BM, we prospectively isolated Lin^–^ SCA-1^+^C-KIT^+^ (LSK) cells (which contain HSCs) and, by genomic PCR, observed complete deletion of *Zeb1* (Figure 1C). Similarly, C-KIT^+^ cells, which constitute both HSCs and committed myeloid and lymphoid progenitors, were fully deleted for *Zeb1* (Figure 1D). In contrast, only partial deletion of *Zeb1* was observed in terminally differentiated T and B cells isolated from the spleen (Figure 1D), suggesting that incomplete deletion observed in BM cells may be ascribed to these cell types.

At 14 days after ablation of *Zeb1*, no significant changes were observed in BM and spleen cellularity or spleen size (Supplemental Figure 1A; supplemental material available online with this article; https://doi.org/10.1172/JCI129115DS1). Immunophenotyping of peripheral blood (PB) revealed a significant reduction in the proportion of MAC1^+^GR1^–^ monocytic cells in *Zeb1^–/–^* mice, while no significant changes were observed in MAC1^+^GR1^+^ cells, which contain granulocytes, or in T cells and B cells in PB or BM (Figure 1E and Supplemental Figure 1B) (26, 27). Intriguingly, despite incomplete deletion of *Zeb1* in lymphoid cells from *Zeb1^–/–^* mice, we observed a selective reduction in CD8^+^ effector memory (CD8^+^ EM) (CD44^hi^CD62L^–^) T cells in PB and BM of *Zeb1^–/–^* mice (Figure 1, F and G). We also found a reduction in CD8^+^ central memory (CD8^+^ CM) (CD44^hi^CD62L^hi^) T cells in spleen of *Zeb1^–/–^* mice, collectively demonstrating a critical role of *Zeb1* in CD8^+^ T cell function (Figure 1H).

### Acute loss of Zeb1 results in a cell-survival defect during thymocyte differentiation and a cell-autonomous T cell differentiation defect.

**Figure 2:**
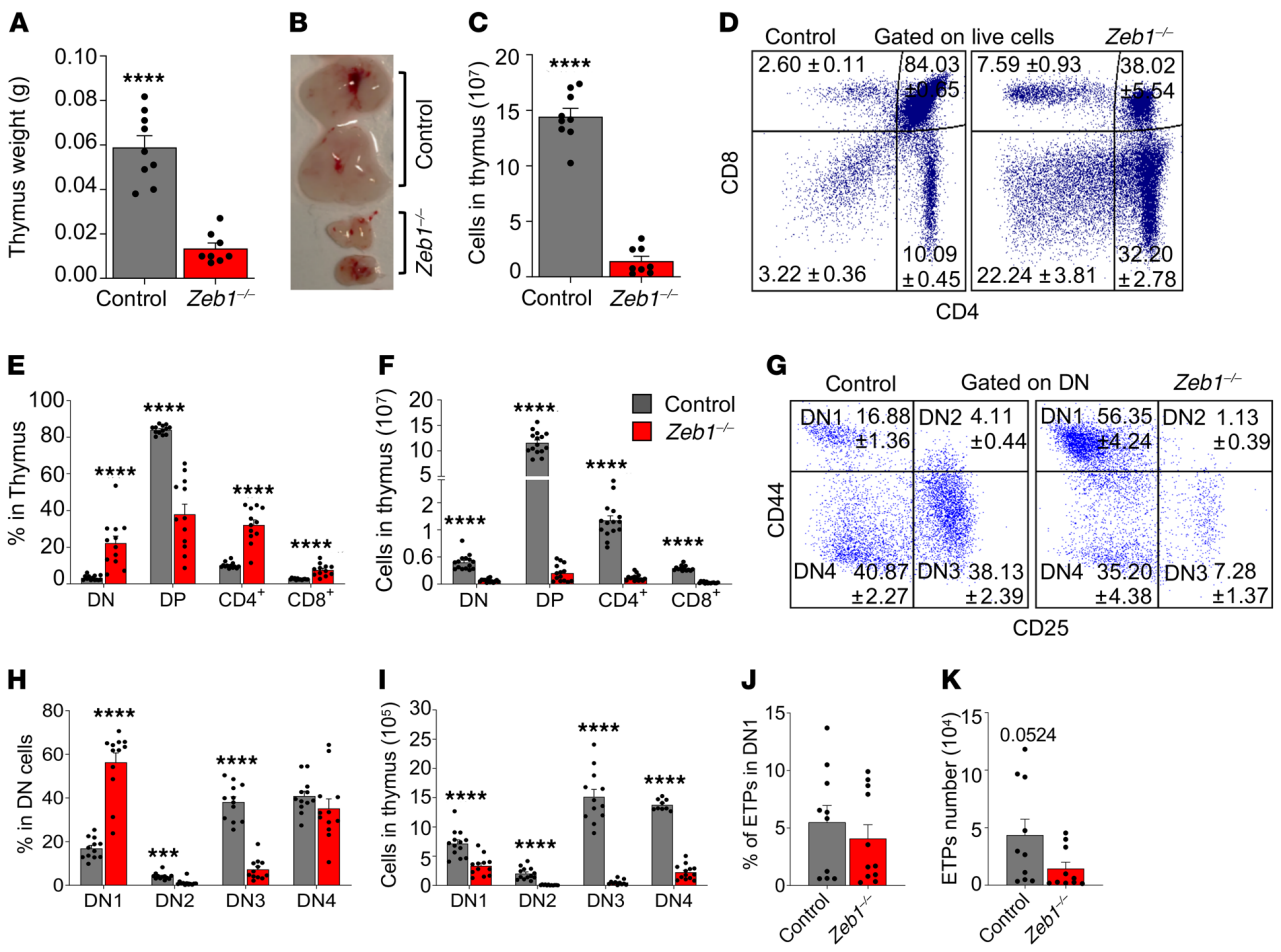
Loss of *Zeb1* results in T cell reduction in thymus associated with early differentiation defects in thymus. Thymus weight (**A**), representative photograph (**B**), and total thymus cellularity (**C**) from control (*n* = 9) and *Zeb1^–/–^* (*n* = 8) mice from 5 independent experiments at day 14 after the last pIpC dose. (**D**) Representative FACS plots of T cell analysis in thymus based on CD4 and CD8 cell-surface markers (DN: CD4^–^CD8^–^, DP: CD4^+^CD8^+^, CD4^+^, CD8^+^). (**E**) Frequency of T cell subsets in thymus from control (*n* = 13) and *Zeb1^–/–^* (*n* = 12) mice from 6 independent experiments at day 14 after the last pIpC dose. (**F**) Total cell count of T cell subsets in thymus from control (*n* = 14–15) and *Zeb1^–/–^* (*n* = 14–15) mice from 7 independent experiments at day 14 after the last pIpC dose. (**G**) Representative FACS plots showing gating strategy of early stages within CD4^+^CD8^+^ DN population using CD25 and CD44 (DN1: CD44^+^CD25^–^, DN2: CD44^+^CD25^+^, DN3: CD44^–^CD25^+^, DN4: CD44^–^CD25^–^) between control and *Zeb1^–/–^* at day 14 after the last pIpC dose. (**H**) Frequency of DN populations (DN1, DN2, DN3, DN4) in DN cells from control (*n* = 12) and *Zeb1^–/–^* (*n* = 12) mice from 5 independent experiments at day 14 after the last pIpC dose. (**I**) Total cell count of DN populations in thymus from control (*n* = 9-13) and *Zeb1^–/–^* (*n* = 11–13) mice from 4 independent experiments at day 14 after the last pIpC dose. (**J**) Frequency and (**K**) total count of ETPs (DN1 cKit^hi^) from control (*n* = 10) and *Zeb1^–/–^* (*n* = 10–11) mice from 4 independent experiments at day 14 after last pIpC dose. Error bars show mean ± SEM. Mann-Whitney *U* test was used to calculate significance. ***P < 0.001; ****P < 0.0001.

Having shown a defect in CD8^+^ T cells in *Zeb1^–/–^* mice and given that germline KO of *Zeb1* results in a developmental defect in the T cell lineage (12, 13), we opted to assess T cell development in the thymus of adult *Zeb1^–/–^* mice. Fourteen days after the last dose of pIpC, *Zeb1^–/–^* mice displayed diminutive thymi coupled with a dramatic reduction in cellularity (Figure 2, A–C). Immunophenotypic analysis of T cell subsets in the thymus revealed an increased frequency of immature double-negative (DN) CD4^–^CD8^–^ cells and mature single-positive (SP) CD4 (CD4^+^) and SP CD8 (CD8^+^) T cells, contrasting with a significant reduction in the proportion of double positive (DP) CD4^+^CD8^+^ cells in *Zeb1^–/–^* mice (Figure 2, D and E). Normalizing for reduced thymic cellularity in *Zeb1^–/–^* mice led to a significant reduction in total cell numbers observed in DN, DP, CD4^+^, and CD8^+^ cells from *Zeb1^–/–^* mice (Figure 2F). This correlated with increased apoptosis in DP, CD4^+^, and CD8^+^ populations, but surprisingly, not in DN cells from *Zeb1^–/–^* mice (Supplemental Figure 1C).

Given that the earliest stages of T cell development were affected by *Zeb1* ablation, we further evaluated the DN cell compartment, which represents the initial stage of thymocyte selection (28, 29). Using CD44 and CD25, the DN population can be subdivided chronologically into 4 populations: DN1 (CD44^+^CD25^–^), DN2 (CD44^+^CD25^+^), DN3 (CD44^–^CD25^+^), and DN4 (CD44^–^CD25^–^) (29), before they become DP cells and ultimately CD4^+^ or CD8^+^ mature cells (29). Immunophenotypic analysis showed increased frequency of DN1 cells and a reduction in DN2 and DN3, while no change was observed in the frequency of DN4 in *Zeb1^–/–^* mice, suggestive of a *Zeb1*-mediated differentiation block in the transition between DN1 and DN2/DN3 (Figure 2, G and H). Analysis of apoptosis in DN subsets revealed increased apoptotic levels in DN2 and DN3 after *Zeb1* deletion, accounting for the differentiation block observed in these compartments, whereas DN1 and DN4 displayed comparable levels of apoptosis between genotypes (Supplemental Figure 1D). When the absolute number of these populations was quantified to account for reduced thymic cellularity of *Zeb1^–/–^* mice, a significant decrease was found across all DN populations following *Zeb1* deletion (Figure 2I).

Next, we evaluated whether the defect observed within immature DN cells was caused by a failure of thymocyte survival preceding selection. We therefore assessed early thymic progenitors (ETPs), characterized as CD4^–^CD8^–^CD44^+^CD25^–^C-KIT^hi^ (30–33). Fourteen days after the last dose of pIpC, we found a comparable frequency of ETPs between control and *Zeb1^–/–^* mice (Figure 2J) with the absolute count of ETPs in *Zeb1^–/–^* thymus showing a near significant reduction compared with control due to reduced thymic cellularity in *Zeb1^–/–^* mice (Figure 2K).

Since the *Mx1-Cre* system deletes genes in nonhematopoietic tissues, such as BM niche cells (25, 34), we assessed whether *Zeb1*-mediated regulation of T cell development was cell autonomous by competitively transplanting CD45.2^+^ BM cells from *Zeb1^fl/fl^;Mx1-Cre^+^* or control mice, admixed with unfractionated CD45.1^+^ BM cells (Supplemental Figure 2A). Six weeks after transplantation, *Zeb1* deletion was induced by injection of pIpC, and 14 days after the last pIpC injection, analysis of donor engraftment in the thymus revealed a dramatic reduction in the *Zeb1^–/–^* genotype compared with control (Supplemental Figure 2B). Consistent with this, a substantial attenuation in donor contribution to DN, DP, CD4^+^, and CD8^+^ cell populations was detected in the *Zeb1^–/–^* genotype (Supplemental Figure 2C). With the exception of ETPs, a significant reduction in donor contribution was found across nearly all DN populations following cell-autonomous *Zeb1* deletion, confirming that *Zeb1* mediates T cell maturation in a cell-autonomous manner (Supplemental Figure 2D). Since mature T cell frequency did not change 14 days after *Zeb1* loss during steady state due to incomplete *Zeb1* deletion (Figure 1D), we also analyzed the donor contribution to peripheral T cells 14 days after *Zeb1* ablation in a cell-autonomous manner, which revealed complete deletion of *Zeb1* and a significant reduction in mature T cells in PB, BM, and spleen (Supplemental Figure 2, E and F). Further, we confirmed that *Zeb1* mediates cell-autonomous reduction in EM CD8^+^ T cells in PB (Supplemental Figure 2G). Together, these data suggest that *Zeb1* is critical for cell survival at the earliest stages of thymocyte differentiation as well as for T cell maturation and maintenance in the thymus. Thus, *Zeb1* is required for cell-autonomous T cell development in the thymus.

### Acute conditional deletion of Zeb1 results in a reduction of lymphoid lineage commitment in BM.

**Figure 3:**
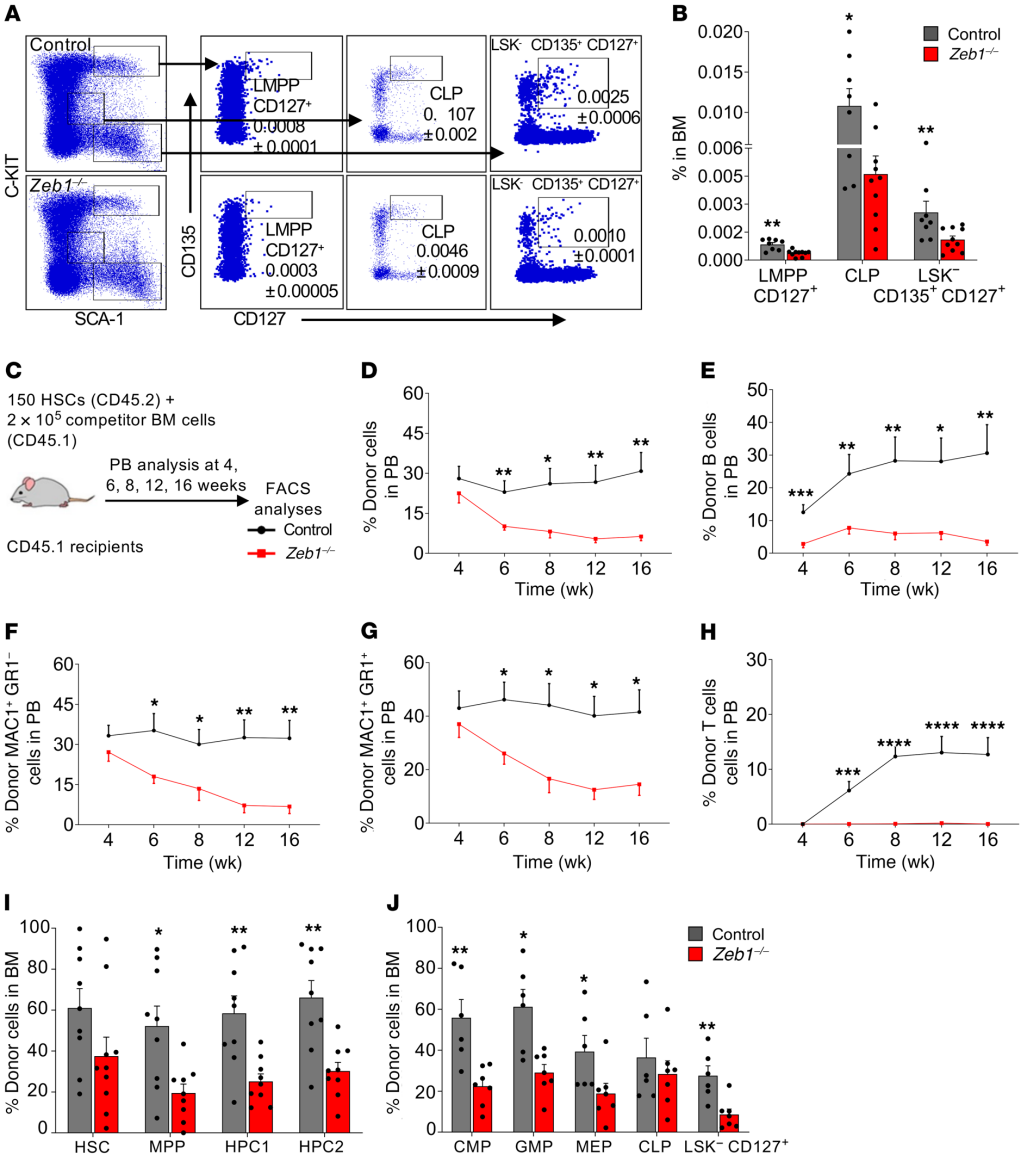
Loss of *Zeb1* results in a reduction of lymphoid progenitors in BM and a multilineage hematopoietic differentiation defect after HSC transplantation. (**A**) Representative FACS plots of the analysis of LMPP CD127^+^ (nonconventional LMPP: LSK CD135^+^CD127^+^), CLP (LIN^–^ SCA-1^lo^C-KIT^lo^CD135^+^CD127^+^), and LSK^–^CD127^+^CD135^+^ 14 days after the last dose of pIpC. (**B**) Frequency of LMPP CD127^+^, CLP, and LSK^–^CD 127^+^CD135^+^ in the BM from control (*n* = 8) and *Zeb1^–/–^* (*n* = 10) mice from 4 independent experiments at day 14 after the last pIpC dose. (**C**) Schematic of competitive HSC transplantation. 150 HSCs from control or *Zeb1^–/–^* mice (donor CD45.2) mixed with 2 × 10^5^ BM competitor cells (CD45.1) were transplanted into lethally irradiated recipients (CD45.1), and the mice were monitored by bleeding the tail vein at different time points until week 16. (**D**) Percentage of donor cells in PB at different time points after HSC transplantation from control (*n* = 10) and *Zeb1^–/–^* (*n* = 10) mice from 3 independent experiments. Analysis of PB donor contribution to B cells (B220^+^) (**E**), MAC1^+^GR1^–^ myeloid cells (**F**), MAC1^+^GR1^+^ myeloid cells (**G**), and T cells (CD4^+^CD8^+^) (**H**) from control (*n* = 10) and *Zeb1^–/–^* (*n* = 8–10) mice from 3 independent experiments. Donor contribution to BM HSPCs (**I**) (HSC: LSK CD150^+^CD48^–^, MPP: LSK CD150^–^CD48^–^, HPC1: LSK CD150^–^CD48^+^, HPC2: LSK CD150^+^CD48^+^) from control (*n* = 9) and *Zeb1^–/–^* (*n* = 9) from 3 independent experiments and BM committed progenitors (**J**) (CMP: LK CD34^+^CD16/32^–^, GMP: CD34^+^CD16/32^+^, MEP: CD34^–^CD16/32^–^, CLP: LIN^–^ SCA-1^lo^C-KIT^lo^CD127^+^, and LSK^–^CD127^+^ from control (*n* = 6) and *Zeb1^–/–^* (*n* = 7) from 2 independent experiments. Error bars show mean ± SEM. Mann-Whitney *U* test was used to calculate significance. *P < 0.05; **P < 0.01; ***P < 0.001; ****P < 0.0001.

We next gauged the impact of *Zeb1* on early T lymphoid lineage commitment in the BM. LSK CD135^hi^CD127^hi^ lympho-myeloid MPPs (LMPPs CD127^+^, nonconventional LMPP) rapidly and efficiently generate T and innate lymphoid cells (35) compared with conventional LMPP (LSK CD34^+^CD135^hi^) (36) or HPC1 (LSK CD150^–^CD48^+^) that overlap functionally with conventional LMPP by 80% (37–39). Interestingly, we found a significant reduction in the proportion of T cell lineage–primed LMPP CD127^+^, but not conventional LMPP, which showed a statistically insignificant trend toward reduction after acute *Zeb1* ablation (Figure 3, A and B, and Supplemental Figure 3A). We assessed other BM lymphoid progenitor compartments, including CLP (LIN^–^SCA-1^lo^C-KIT^lo^CD127^hi^CD135^hi^) (35, 40) and LIN^–^ SCA-1^+^C-KIT^–^ (LSK^–^CD135^+^CD127^+^) (41) that were reduced in *Zeb1^–/–^* mice (Figure 3, A and B). Together, these data suggest that *Zeb1* acts as a critical modulator of incipient lymphoid progenitor commitment from HSCs.

### Acute conditional deletion of Zeb1 in HSCs results in a profound self-renewal and multilineage hematopoietic differentiation defect.

To directly assess the impact of acute deletion of *Zeb1* in HSCs, we performed flow cytometry analysis on immunophenotypically defined HSCs and all MPP populations from control or *Zeb1^–/–^* mice. The frequency of HSCs (LSK CD150^+^CD48^–^) and MPPs (LSK CD150^–^CD48^–^) was comparable between control and *Zeb1^–/–^* genotypes (Supplemental Figure 3B). HPC1 (LSK CD150^–^CD48^+^) showed a nonsignificant reduction after *Zeb1* deletion similar to that observed in conventional LMPP (LSK CD34^+^CD135^+^) (Supplemental Figure 3, A and B). *Zeb1^–/–^* HPC2 (LSK CD150^+^CD48^+^), which possesses both myeloid and lymphoid potential (38), showed a significant reduction in the frequency in total BM compared with control (Supplemental Figure 3B). These data demonstrate that *Zeb1* regulates the abundance of select MPP populations.

Having observed a reduction of MAC1^+^GR1^–^ myeloid cells in PB following acute deletion of *Zeb1* in the hematopoietic system, we asked whether this was due to defects in committed myeloid progenitors from BM *Zeb1^–/–^*. No significant difference in CMP (LK CD34^+^CD16/32^–^), GMP (LK CD34^+^CD16/32^+^), and MEP (LK CD34^–^CD16/32^–^) populations was noted between control and *Zeb1^–/–^* mice (Supplemental Figure 3C). Thus, *Zeb1*-mediated regulation of terminal MAC1^+^GR1^–^ myeloid cell differentiation appears to be independent of committed myeloid progenitor maturation from BM.

To stringently test the functionality and differentiation capacity of HSCs from *Zeb1*^–/–^ mice, we prospectively isolated 150 HSCs (CD45.2) from control or *Zeb1*^–/–^ mice at 14 days following deletion, mixed them with 2 × 10^5^ BM competitor cells (CD45.1), and transplanted this cell preparation into lethally irradiated recipients (CD45.1) (Figure 3C). The engraftment capacity of transplant recipients in PB was monitored until week 16 (Figure 3C). Significant engraftment failure was observed by week 6 and continued to decrease progressively until week 16 (Figure 3D). To test the donor contribution to PB of specific hematopoietic lineages, we analyzed PB for CD45.2 (donor) and CD45.1 (competitor) in conjunction with MAC1^+^GR1^–^ myeloid, MAC1^+^GR1^+^ myeloid, B220^+^ B cells, and CD4^+^CD8^+^ T cells. A profound reduction in donor contribution to B cells (Figure 3E), MAC1^+^GR1^–^ myeloid cells (Figure 3F), and MAC1^+^GR1^+^ myeloid cells (Figure 3G) was observed in recipients of *Zeb1^–/–^* HSCs. No engrafted T cells were derived from recipients transplanted with *Zeb1^–/–^* HSCs (Figure 3H).

Having observed multilineage defects in terminally differentiated blood cells in recipients of *Zeb1^–/–^* HSCs, we asked whether these defects originated in parental HSPCs or lineage-committed progenitors. Within LSK compartments, the donor contribution to HSC was equal between recipients of control or *Zeb1*^–/–^ HSCs (Figure 3I). However, there was a significant reduction in the donor contribution to MPP, HPC1, and HPC2 in *Zeb1*^–/–^ compared with control (Figure 3I). We also analyzed committed progenitors downstream of HSPCs and found a dramatic reduction in donor contribution to CMP, GMP, MEP, and LSK^–^CD127^+^, but no change was observed in donor contribution to CLP (Figure 3J). These data directly link the functional defects observed after transplantation of *Zeb1*-deficient HSCs to alterations in specific HSPC and lineage committed progenitor compartments.

An integral part of successful engraftment after BM transplantation is homing of i.v. infused HSPCs to the BM niche, the main home of adult hematopoiesis. To assess whether the *Zeb1*^–/–^ engraftment defect was due to abnormal homing of *Zeb1^–/–^* HSPCs to the BM, we transplanted 7 × 10^6^ total BM cells (CD45.2) from control or *Zeb1*^–/–^ mice into lethally irradiated recipients (CD45.1) and analyzed donor cell presence in recipients at 18 hours after transplantation (Supplemental Figure 4A). Relative parity was observed in the homing capacity of total BM cells or LSK populations in the 2 genotypes (Supplemental Figure 4B). Similarly, homing of donor cells to the spleen and thymus was comparable between control and *Zeb1*^–/–^ genotypes (Supplemental Figure 4C). Thus, acute deletion of *Zeb1* does not impact the homing ability of hematopoietic cells in vivo.

**Figure 4:**
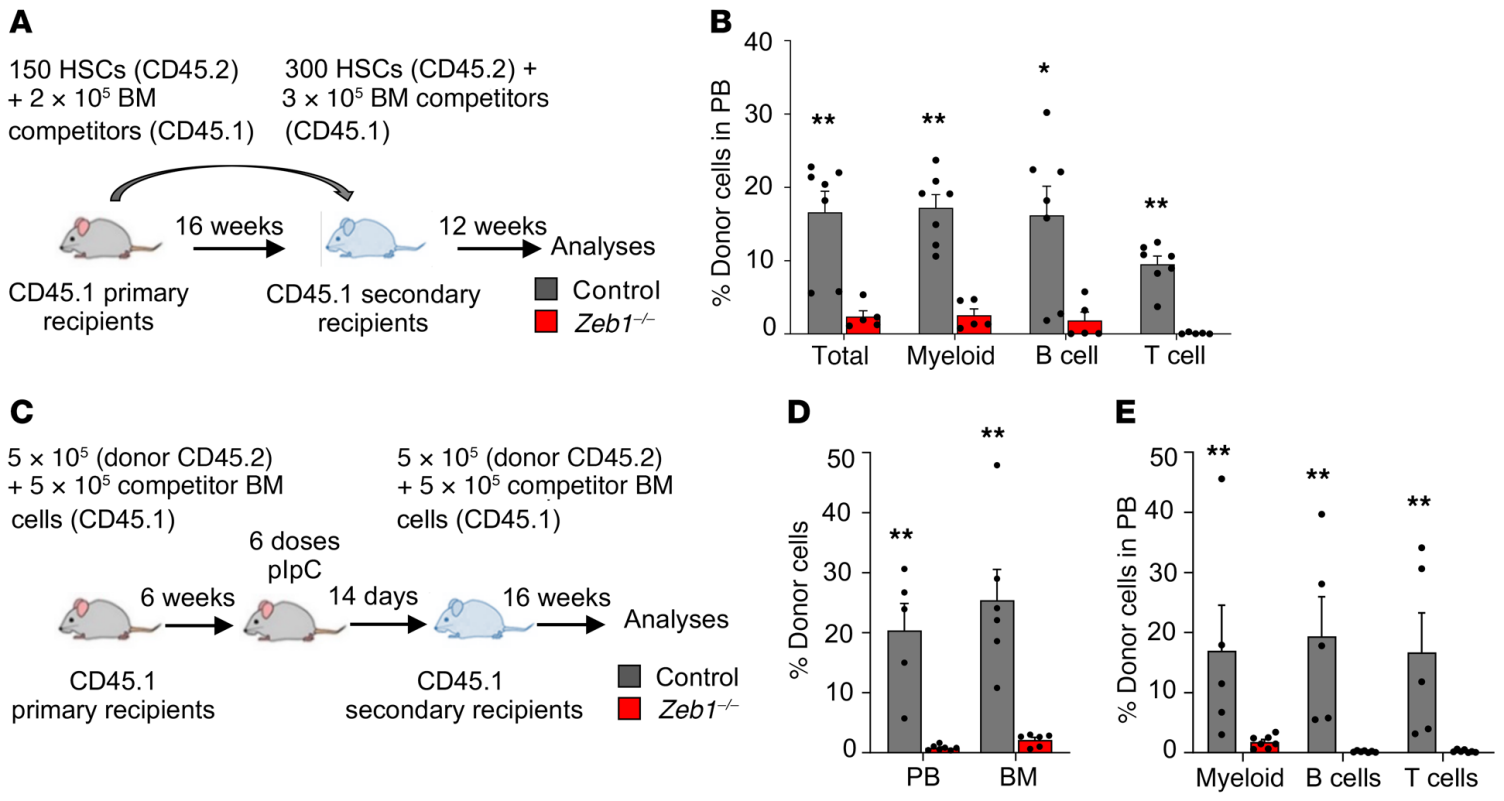
*Zeb1* regulates HSC self-renewal and differentiation in a cell-autonomous manner. (**A**) Schematic of secondary HSC transplantation. 300 CD45.2^+^ HSCs from primary recipients from control or *Zeb1^–/–^* mice mixed with 3 × 10^5^ BM competitor cells (CD45.1) were transplanted into lethally irradiated recipients (CD45.1), and the mice were analyzed at week 12. (**B**) Percentage of donor cells in PB and donor contribution to myeloid (MAC1^+^), B (B220^+^), and T (CD4^+^/CD8^+^) cells at week 12 after secondary HSC transplantation from control (*n* = 7) and *Zeb1^–/–^* (*n* = 5) from 2 independent experiments. (**C**) Schematic of the secondary total BM transplantation in cell-autonomous manner. 5 × 10^5^ CD45.2^+^ BM cells from primary recipients 14 days after the last pIpC dose from control or *Zeb1^–/–^* mice mixed with 5 × 10^5^ BM competitor cells (CD45.1) were transplanted into lethally irradiated recipients (CD45.1), and the mice were analyzed at week 16. (**D**) Percentage of donor cells in PB and BM at week 16 after secondary cell autonomous total BM transplantation from control (PB *n* = 5, BM = 6) and *Zeb1^–/–^* (PB *n* = 7, BM = 6) mice from 1 experiment. (**E**) Donor contribution to PB MAC1^+^ myeloid cells, B220^+^ B cells, and CD4^+^/CD8^+^ T cells at week 16 after secondary cell-autonomous total BM transplantation from control (*n* = 5) and *Zeb1^–/–^* (*n* = 7) mice from 1 experiment. Error bars show mean ± SEM. Mann-Whitney *U* test was used to calculate significance. *P < 0.05; **P < 0.01.

To directly test the impact of *Zeb1* deletion on the self-renewal capacity of HSCs, we performed secondary transplantation of *Zeb1^–/–^* HSCs. We sorted 300 HSCs (CD45.2) from control or *Zeb1^–/–^* primary recipients and admixed them with 3 × 10^5^ competitor BM cells before transplanting them into lethally irradiated recipients. We observed a strong defect in PB engraftment associated with multilineage hematopoietic impairment in secondary transplant recipients by week 12 (Figure 4, A and B), indicative of a self-renewal defect in *Zeb1^–/–^* HSCs.

### Zeb1 is required for cell-autonomous HSC function.

To assess whether the acute requirement for *Zeb1* in maintaining HSC function was cell autonomous, we performed a competitive BM transplantation by transplanting 5 × 10^5^ BM cells from control and *Zeb1*^fl/fl^*;Mx1-Cre*^+^ (CD45.2) mice admixed with equal number of WT competitor cells (CD45.1) into lethally irradiated recipients (CD45.1). Six weeks later, *Zeb1* deletion was induced by administering recipients with pIpC,and 14 days after the last dose of pIpC, mice were sacrificed and 5 × 10^5^ donor BM cells (CD45.2) from primary recipients (14 days after *Zeb1* ablation) mixed with 5 × 10^5^ competitor BM cells (CD45.1) were transplanted into lethally irradiated recipients (Figure 4C). Sixteen weeks after transplantation, we found a dramatic reduction in donor engraftment in PB and BM (Figure 4D). Further analysis of donor contribution to PB lineages revealed a marked reduction in myeloid cells and near loss of B and T cells (Figure 4E).

To determine whether the Zeb1^–/–^ BM microenvironment plays a role in Zeb1-mediated HSC regulation, we transplanted 1 × 10^6^ WT BM cells (CD45.1) into lethally irradiated control or Zeb1^fl/fl^;Mx1-Cre^+^ (CD45.2) mice. Six weeks later, we injected the recipients with pIpC to delete *Zeb1* and analyzed the mice at week 16 after the last dose of pIpC (Supplemental Figure 4D). PB analyses of myeloid cells (MAC1^+^), B cells (B220^+^), and T cells (CD4^+^/CD8^+^) showed no significant difference between control and *Zeb1^–/–^* (Supplemental Figure 4E). Next, we asked whether the altered BM niche would affect the composition of HSPC and committed progenitors. The data showed that the frequency of these populations was comparable between control and *Zeb1^–/–^* (Supplemental Figure 4, F and G). Together, these data demonstrate that *Zeb1* is required for cell-autonomous HSC functionality.

### Zeb1^–/–^ HSCs display deregulated cell polarity and hematopoietic differentiation transcriptional signatures.

**Figure 5:**
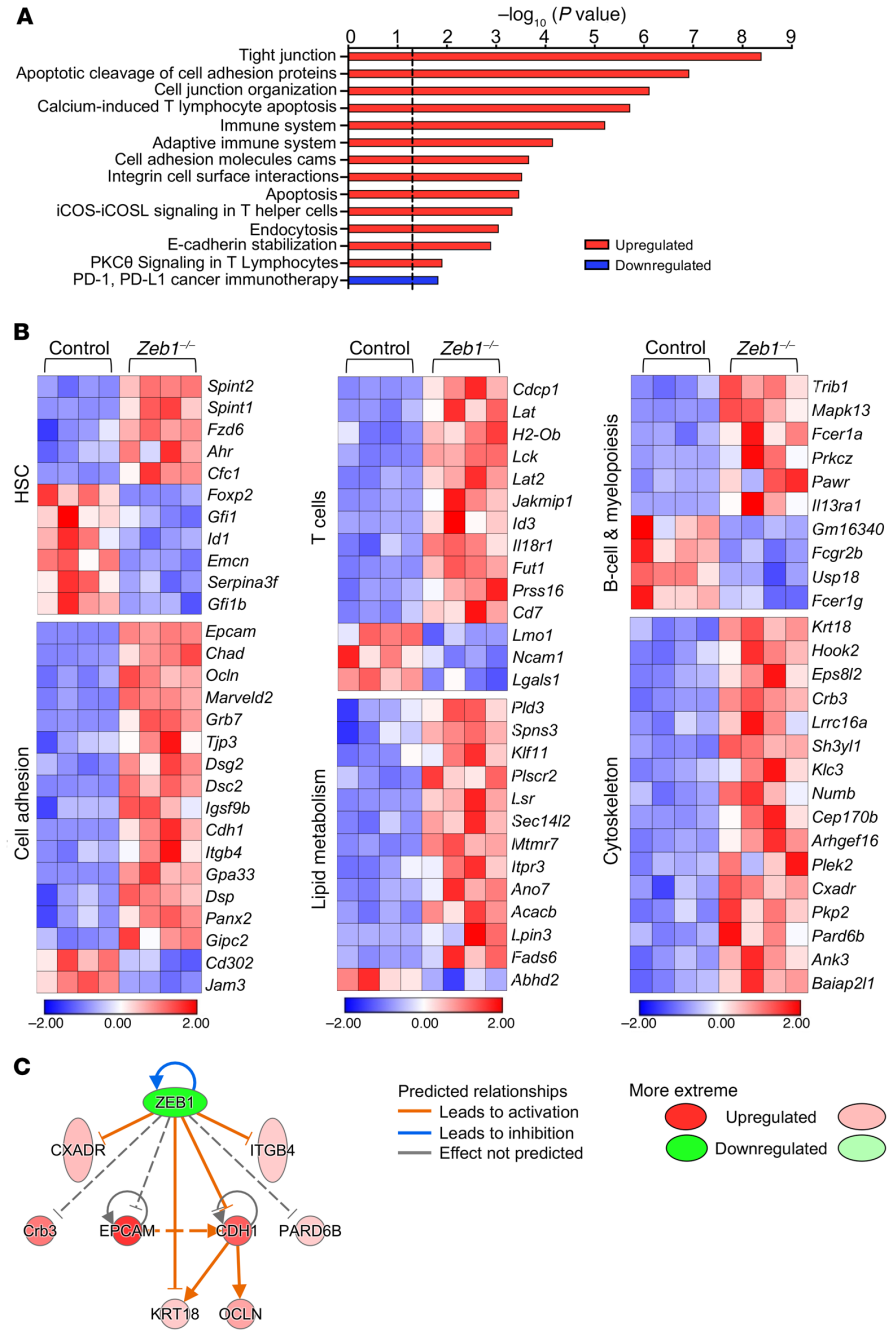
*Zeb1^–/–^* HSCs display deregulation of hematopoietic function and cell polarity transcriptional programming. RNA-Seq was performed in sorted control and *Zeb1^–/–^* HSCs (LSK CD150^+^CD48^–^) 14 days after last pIpC dose (*n* = 4 for each genotype). (**A**) Biological pathway analysis shows the top enriched pathways in *Zeb1^–/–^* HSCs compared with control. Data are shown as –log_10_ (*P* value), and the dashed black line indicates *P* value of 0.05. (**B**) Heatmaps of the DEGs after *Zeb1* deletion related to HSC function, T cells, and B cells as well as cytoskeleton, lipid metabolism, and cell adhesion. Heatmap scale represents *z* score. (**C**) A network of *Zeb1* interaction with several target genes related to polarity, cytoskeleton, and cell adhesion using IPA software. Due to their confirmed binding to ZEB1 in the literature, Epcam, Pard6b, and Crb3 were added manually.

**Figure 6:**
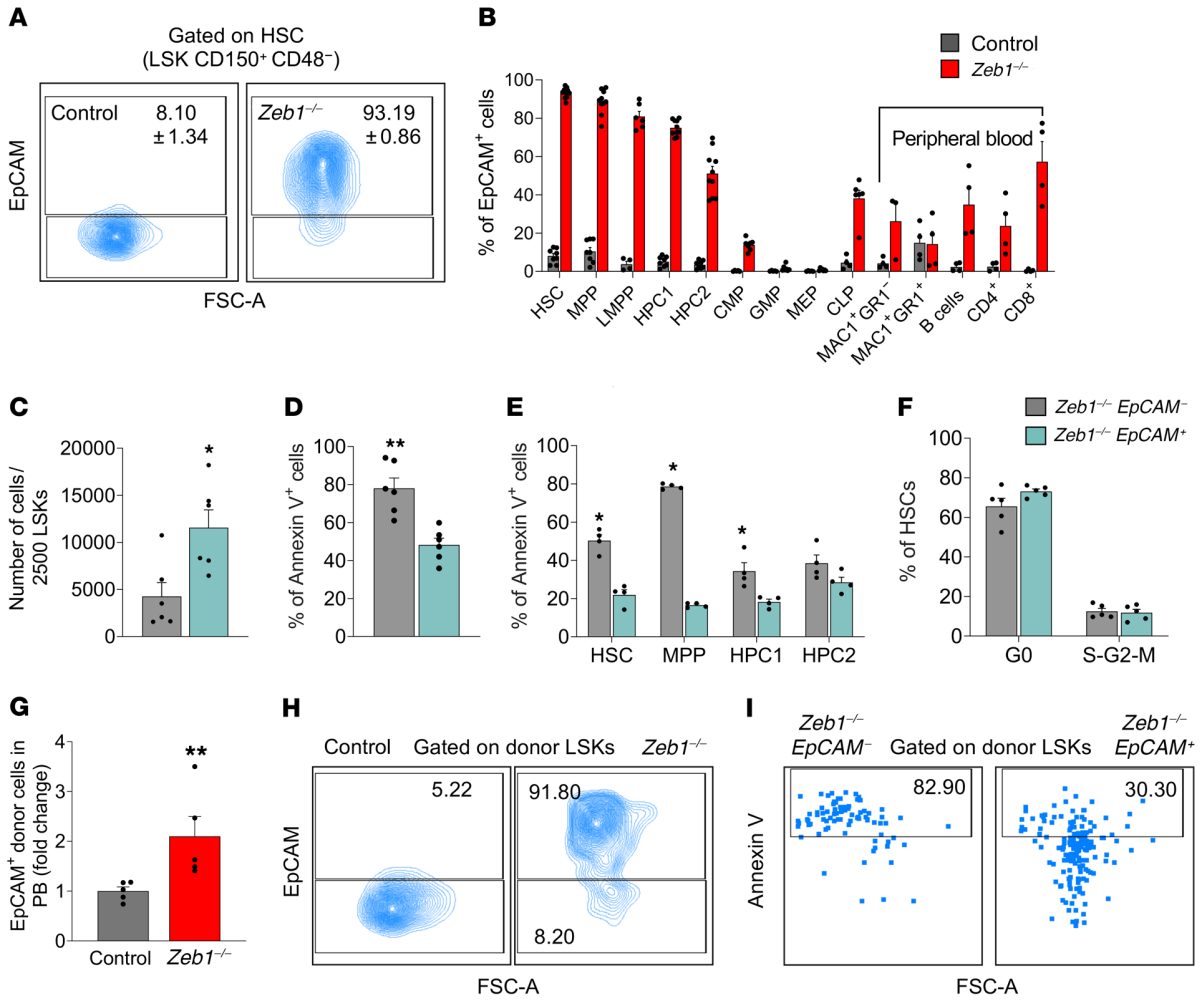
Increased EpCAM expression confers survival advantage and differentiation block in *Zeb1^–/–^* HSCs. (**A**) Representative flow cytometry plots of EpCAM expression in HSCs 14 days after pIpC injection. (**B**) Analysis of EpCAM expression in BM subpopulations and PB mature cells 14 days after pIpC injection from control (*n* = 8 for HSC, MPP, HPC1, and HPC2; *n* = 4 for LMPP, CLP and mature PB populations; *n* = 5 for CMP, GMP, and MEP) and *Zeb1^–/–^* (*n* = 10 for HSC, MPP, HPC1, and HPC2; *n* = 6 for LMPP and CLP; *n*= 4 for mature PB populations except MAC1^+^GR1^–^ n = 3; n = 7 for CMP, GMP, and MEP). (**C**) Cell number after culturing 2500 LSKs from *Zeb1^–/–^ EpCAM^–^* (*n* = 6) and *Zeb1^–/–^ EpCAM^+^* (*n* = 6) from 3 independent experiments. (**D**) Analysis of apoptosis in LSKs after culture from *Zeb1^–/–^ EpCAM^–^* (*n* = 6) and *Zeb1^–/–^ EpCAM^+^* (*n* = 6) from 3 independent experiments. (**E**) Analysis of apoptosis in fresh BM HSPCs 14 days after pIpC injection from *Zeb1^–/–^ EpCAM^–^* (*n* = 4) and *Zeb1^–/–^ EpCAM^+^* (*n* = 4) from 2 independent experiments. (**F**) Cell cycle analysis of HSCs using Ki67 and DAPI 14 days after pIpC injection from *Zeb1^–/–^ EpCAM^–^* (*n* = 5) and *Zeb1^–/–^ EpCAM^+^* (*n* = 5) from 1 experiment. (**G**) Analysis of EpCAM expression in donor PB at week 16 after primary HSC transplantation from control (*n* = 5) and *Zeb1^–/–^* (*n* = 5) from 1 experiment represented as fold change. (**H**) Representative FACS plots of the analysis of EpCAM expression in LSKs 16 weeks after primary HSC transplantation from control (*n* = 2) and *Zeb1^–/–^* (*n* = 1) from 1 experiment. (**I**) Representative FACS plots of the analysis of apoptosis using annexin V in EpCAM-negative and -positive fractions within donor LSKs 16 weeks after primary HSC transplantation from control (*n* = 2) and *Zeb1^–/–^* (*n* = 1) from 1 experiment. Error bars show mean ± SEM. Mann-Whitney *U* test was used to calculate significance. *P < 0.05; **P < 0.01.

In order to understand the transcriptional signature underpinning *Zeb1*-mediated regulation in HSCs, we performed RNA-Seq on purified HSCs (LSK CD150^+^CD48^–^) from control or *Zeb1^–/–^* mice 14 days after the last dose of pIpC. Of 222 differentially expressed genes (DEG), 47 genes (21%) were downregulated and 175 upregulated (79%) from *Zeb1^–/–^* HSCs. These data are largely consistent with the notion that *Zeb1* functions as a transcriptional repressor (42). Biological pathway analysis confirmed that the most enriched pathways were upregulated and included tight junction, cell adhesion, cell junction organization, immune system, and endocytosis pathways (Figure 5A). *Zeb1* appears to regulate a transcriptional signature related to cell polarity, consisting of genes related to cytoskeleton, cell adhesion, and lipid metabolism/lipid membrane biology (Figure 5B), congruent with the idea that *Zeb1* acts as a potent inducer of the EMT process, involving *Zeb1*-mediated repression of cell polarity genes (43–45). Using Ingenuity Pathway Analysis software (IPA), we generated a gene-interaction network showing the direct regulation of *Zeb1*-specific target genes related to cell polarity, cytoskeleton, and cell adhesion that included a regulatory node involving epithelial cell adhesion molecule (EpCAM), CRB3, PARD6b, ITGB4, CDH1, KRT18, and OCLN (10, 43–50) (Figure 5C). In agreement with this transcriptional network, EpCAM, CDH1, and ITGB4 upregulation in *Zeb1^–/–^* HSPCs was confirmed at the protein level by flow cytometry (Figure 6, A and B, and Supplemental Figure 5, A and B). Reflecting the global, multilineage differentiation functional defects of *Zeb1*^–/–^ HSCs, we observed a broad, robust pattern of deregulated HSC maintenance and hematopoietic lineage affiliated from both myeloid and lymphoid lineages (Figure 5B).

With prominent transcriptional deregulation of T cell pathways being observed in *Zeb1*^–/–^ HSCs (Figure 5A), we next asked whether the defects in *Zeb1*^–/–^ HSCs could be associated with those observed in *Zeb1*^–/–^ T cells. To address this question at the transcriptional level, we conducted RNA-Seq from *Zeb1*^–/–^ or control CLPs, which have T lymphoid but not myeloid potential, and compared their transcriptional signatures with that from *Zeb1*^–/–^ HSCs. *Zeb1*^–/–^ CLPs displayed gene expression pathways comparable to those of *Zeb1*^–/–^ HSCs, including deregulated cell-cell junction, tight junction, cell adhesion, cytoskeleton, and T cell pathways (Supplemental Figure 5, C–E). Remarkably, of the 47 DEGs in *Zeb1*^–/–^ CLPs, 27 genes (57%) were also differentially expressed in *Zeb1*^–/–^ HSCs (Supplemental Figure 5F). Other biological pathways reflecting T cell function were deregulated in *Zeb1*^–/–^ HSCs but not *Zeb1*^–/–^ CLPs (e.g., calcium-induced T lymphocyte apoptosis, iCOS-iCOSL signaling in T helper cells) (Figure 5A). Transcriptional signatures relating to the CtBP1 pathway (51, 52) were observed only in *Zeb1*^–/–^ CLPs (Supplemental Figure 5C). However, the majority of transcriptional programing mediating the differentiation defect of *Zeb1*^–/–^ T cells was instigated in HSCs and transmitted to CLPs. Overall, in its capacity as a transcriptional repressor, *Zeb1* acts as a potent regulator of cell polarity and differentiation-affiliated transcriptional signatures in HSCs.

### Increased EpCAM expression confers a cell-survival advantage in Zeb1^–/–^ HSCs that alters self-renewal and differentiation fates.

EpCAM, a glycoprotein mediating cell adhesion in epithelia (53), was the most highly upregulated gene in *Zeb1^–/–^* HSCs (Figure 5B). In other types of stem cells, EpCAM has been established as a crucial regulator of stem cell maintenance and differentiation (46). We therefore elected to examine the impact of EpCAM expression in the context of *Zeb1*-mediated regulation of HSC fate. We first confirmed enhanced expression of EpCAM at the protein level by flow cytometry of *Zeb1^–/–^* HSC, MPP, HPC1, and HPC2 populations (Figure 6, A and B). While EpCAM expression was nearly extinguished during differentiation to CMP, GMP, and MEP committed progenitors, it was upregulated in terminally differentiated cells (myeloid, B and T lineages) from PB (Figure 6B). EpCAM-positive HSPCs from *Zeb1^–/–^* LSKs expanded more than their EpCAM-negative counterparts in vitro (Figure 6C), suggesting that EpCAM expression confers a cell-survival signal in *Zeb1^–/–^* HSCs. We directly addressed whether EpCAM expression mediates cell survival in freshly isolated *Zeb1^–/–^* HSCs and in vitro and observed reduced apoptosis in *Zeb1^–/–^* *EpCAM^+^* HSCs and MPP subsets in both settings (Figure 6, D and E). Cell cycle status based on EpCAM expression was unperturbed in *Zeb1^–/–^* HSCs (Figure 6F). By evaluating the impact of EpCAM expression in *Zeb1^–/–^* HSC survival and differentiation in vivo, we showed at 16 weeks after HSC transplantation that *Zeb1^–/–^* cells in PB displayed a 2-fold increase in EpCAM expression compared with controls (Figure 6G). Thus, EpCAM expression in *Zeb1^–/–^* HSC correlates with the multilineage differentiation block observed during transplantation (Figure 3 and Figure 4). Yet high expression of EpCAM was preserved in *Zeb1^–/–^* HSPCs at 16 weeks after transplant and these *EpCAM^+^* *Zeb1^–/–^* HSPCs had a lower propensity for apoptosis (Figure 6, H and I). Together, these data suggest that augmented EpCAM expression confers a cell survival advantage in *Zeb1^–/–^* HSCs that causes an imbalance between self-renewal and differentiation fates.

### Zeb1^–/–^ EpCAM^+^ HSPCs display enhanced cell survival and diminished mitochondrial metabolism, RNA biogenesis, and differentiation transcriptional signatures.

**Figure 7:**
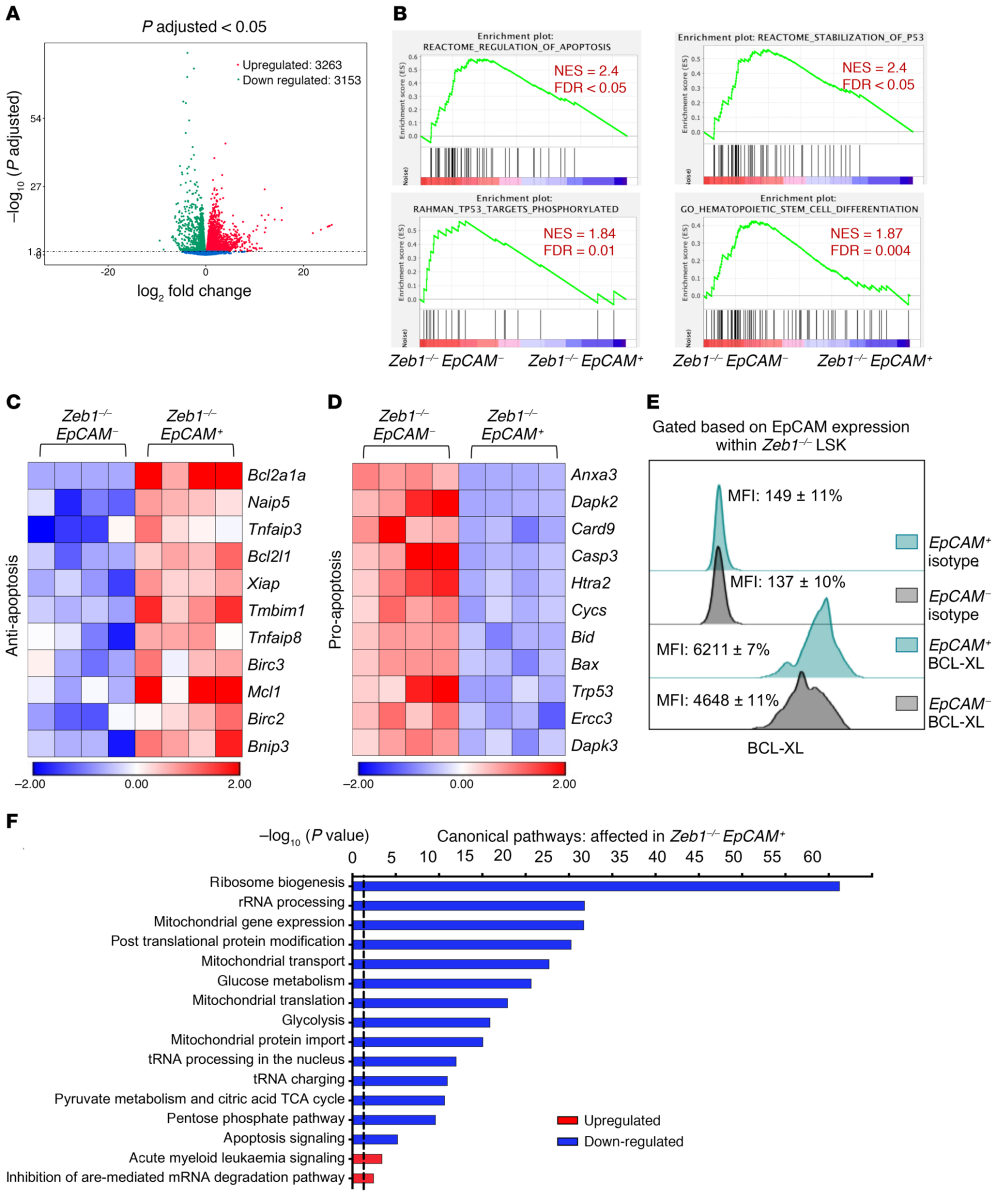
*Zeb1^–/–^ EpCAM^+^* HSPCs display enhanced cell survival and diminished mitochondrial metabolism, RNA biogenesis, and differentiation transcriptional signatures. (**A**) Volcano plot showing the relationship between magnitude of gene expression change (log_2_ of fold-change; *x* axis) and statistical significance of this change (–log_10_ of adjusted *P* value; *y* axis) in a comparison of *Zeb1^–/–^ EpCAM^+^* to *Zeb1^–/–^ EpCAM*^–^ LSK cells. Colored points represent DEGs (cutoff FDR < 0.05) that are either overexpressed (red) or underexpressed (green) in *Zeb1^–/–^* *EpCAM^+^* compared with *Zeb1^–/–^ EpCAM^–^*. (**B**) GSEA plots of regulation of apoptosis, stabilization of P53, TP53 targets phosphorylated, and HSC differentiation. Heatmaps of the DEGs within *EpCAM^+^* and *EpCAM^–^* LSK after *Zeb1* deletion related to antiapoptosis (**C**) and proapoptosis (**D**). (**E**) Representative histogram of BCL-XL levels in EpCAM fractions within *Zeb1^–/–^* LSK. (**F**) Canonical pathways that were mostly enriched in *Zeb1^–/–^ EpCAM^+^* LSK cells derived from the IPA, BioCarta, KEGG, PID, and Reactome pathway databases. Data are shown as –log_10_ (*P* value), and the dashed black line indicates *P* value of 0.05. Analysis was performed using the GSEA software and IPA.

To evaluate the transcriptomic signature demarcating *Zeb1^–/–^ EpCAM^+^* HSPCs from *Zeb1^–/–^ EpCAM^–^* HSPCs, we performed RNA-Seq on *Zeb1^–/–^ EpCAM^+^* LSK cells or *Zeb1^–/–^ EpCAM^–^* LSK cells 14 days after the last dose of pIpC. In *Zeb1^–/–^ EpCAM^+^* HSPCs, 3263 genes were upregulated and 3153 genes were downregulated (Figure 7A). In agreement with enhanced cell survival and a functional block in differentiation associated with *Zeb1^–/–^ EpCAM^+^* HSCs (Figure 6), biological pathway analysis revealed a robust p53-mediated prosurvival signature and an antihematopoietic differentiation signature in *Zeb1^–/–^* *EpCAM^+^* HSPCs (Figure 7, B–D). Furthermore, we observed augmented expression of antiapoptotic BCL-XL (54) in *Zeb1^–/–^ EpCAM^+^* HSPCs (Figure 7, C and E) and an EpCAM-p53-BCL-XL (BCL2L1) interacting gene network of apoptotic regulation in *Zeb1^–/–^* HSPCs (Supplemental Figure 6).

Mitochondria play crucial regulatory roles in fundamental cellular processes, such as apoptosis and bioenergetic provisions (55), and in the context of HSCs, act as a gatekeeper limiting HSC self-renewal ability (56). *Zeb1^–/–^ EpCAM^+^* HSPCs displayed reduced mitochondrial gene expression, transport, translation, and protein import as well as reduced associated mitochondrial metabolic pathways (e.g., pyruvate metabolism and TCA cycle) critical to HSC fate (57) (Figure 7F). Further highlighting the relatively low bioenergetic state of *Zeb1^–/–^ EpCAM^+^* HSPCs, ribosome biogenesis and ribosome-associated pathways, such as rRNA processing, whose reduction has previously been associated with conferral of stress resistance in preleukemic HPSCs (58), were similarly downregulated in *Zeb1^–/–^ EpCAM^+^* HSPCs (Figure 7F). Consistent with this, *Zeb1^–/–^ EpCAM^+^* HSPCs also displayed augmented AML signaling (Figure 7F). Therefore, in addition to control of cell survival, *Zeb1*-mediated repression of EpCAM appears to be critical in regulating mitochondrial metabolism and ribosomal pathways associated with normal HSC maintenance and prevention of preleukemic and leukemic signaling.

### ZEB1 expression is downregulated in AML patients, and acute deletion of Zeb1 in leukemic stem cells enhances disease progression in MLL-AF9 and Meis1a/Hoxa9 driven AML.

**Figure 8:**
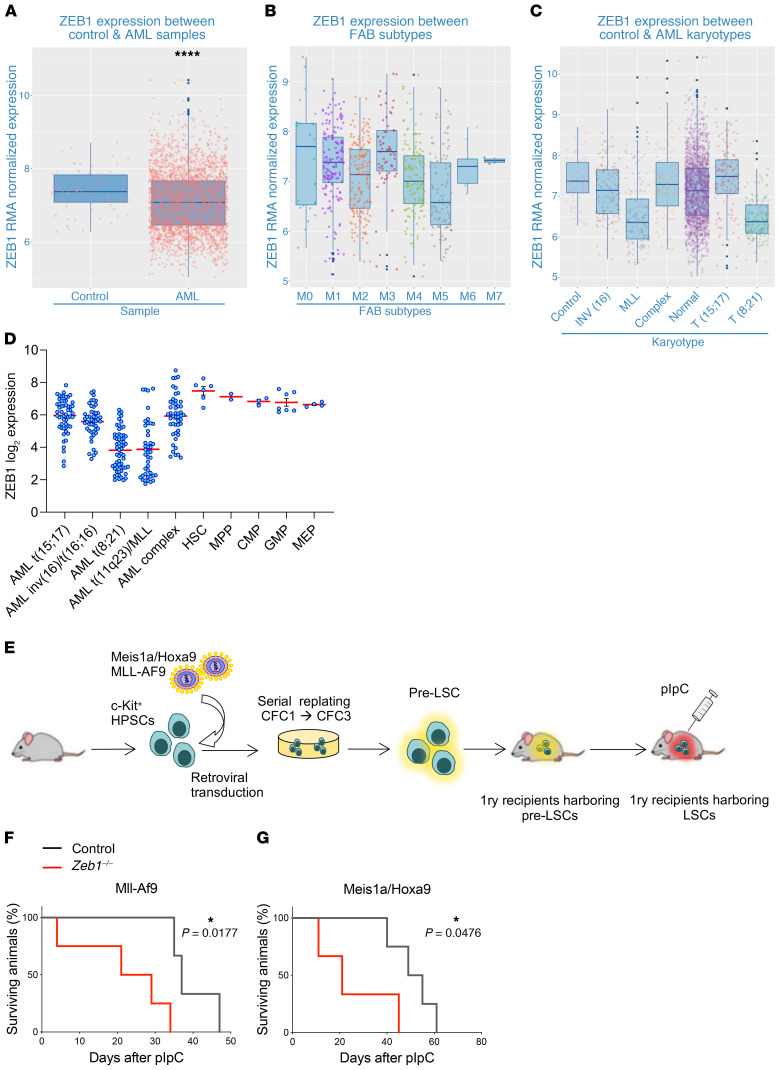
*Zeb1* is downregulated in AML patient samples and acts as a tumor suppressor in MLL-AF9 and Meis1a/Hoxa9-driven AML. (**A**–**C**) ZEB1 RMA–normalized expression from combined published data sets in (**A**) control and AML, (**B**) across FAB subtypes and, (**C**) karyotypes. (**D**) ZEB1 log_2_ expression data in human HSPC and AML karyotypes. Data from BloodSpot. Error bars show mean ± SEM. Student’s *t* test was used unless otherwise indicated. ****P < 0.0001. (**E**) C-KIT^+^ cells from control and *Zeb1^fl/fl^;Mx1-Cre^+^* mice were transduced with retroviruses expressing MLL-AF9 or Meis1a/Hoxa9 oncogenes and plated into CFC assays. After CFC3 (6 days each CFC), pre-LSCs (CD45.2^+^C-KIT^+^) were sorted and transplanted into lethally irradiated recipients together with CD45.1^+^ unfractionated BM cells. Three weeks later, mice were administered pIpC to induce gene deletion and monitored for AML development. (**F** and **G**) Kaplan-Meier survival curve of primary recipients transplanted with (**F**) MLL-AF9 (*n* = 4) or (**G**) Meis1a/Hoxa9 (*n* = 4) pre-LSCs. Mantel-Cox test. *P < 0.05.

Subversion of HSC fates may cause hematologic neoplasia, including leukemia (59). Having found that *Zeb1* deficiency leads to critical impairments in HSC self-renewal, apoptotic, and differentiation fates and because increased EpCAM expression in *Zeb1-*deficient HSCs enhanced AML signaling, we assessed the role of *Zeb1* in AML. We initially evaluated ZEB1 expression in a large cohort of AML patients. AML (*n* = 2611) and control (*n* = 77) patient data sets were obtained from NCBI’s Gene Expression Omnibus (GEO) to assemble a case/control cohort hybridized to Affymetrix Human Genome U133 Plus 2.0 GeneChip array and analyzed through R using bioconductor packages, where data was normalized using Robust Multi-Array Average (RMA). We observed that ZEB1 expression was lower in AML patients compared with healthy controls (Figure 8A). Attenuated expression of ZEB1 was particularly prevalent in M4 and M5 FAB subtypes and also in AML patients with t(8;21) and *MLL* chromosomal translocations (60, 61) (Figure 8, B and C). Using an independent AML patient database, BloodSpot (http://www.bloodspot.eu), we validated lower ZEB1 expression in patients harboring these chromosomal translocations (Figure 8D).

While these data imply that *ZEB1* acts as a tumor suppressor in AML, the functional requirement of *Zeb1* in AML disease progression remains unknown. To directly assess this, we employed an assay in which leukemic transformation of murine C-KIT^+^ HSPCs is mediated by retroviral overexpression of either *MLL-AF9* or *Meis1a/Hoxa9* AML oncogenes (62–64). *MLL-AF9*– or *Meis1a/Hoxa9*-transduced cells were serially passaged for 3 rounds in colony-forming cell (CFC) assays to generate pre–leukemic stem cells (pre-LSCs), which, on i.v. injection into primary lethally irradiated mice, become LSCs — the malignant counterparts of HSCs that drive disease progression in AML (65) (Figure 8E). We cotransduced HSPCs from noninduced *Zeb1^fl/fl^;Mx1-Cre*^+^ or control mice with retroviruses expressing either *MLL-AF9* or *Meis1a/Hoxa9*, collected pre-LSCs, and transplanted them into primary recipients alongside unfractionated BM support cells (Figure 8E). By flow cytometry, we assessed the PB of recipients for engrafting leukemic cells and induced *Zeb1* deletion with pIpC after disease onset, when 20% engraftment of leukemic cells was observed in the PB (66) (data not shown). In both *MLL-AF9* and *Meis1a/Hoxa9* leukemic models, recipients of *Zeb1*-KO LSCs succumbed to AML with enhanced rapidity compared with recipients receiving control LSCs, indicating that *Zeb1* deletion accelerates LSC-mediated disease progression (Figure 8, F and G). Thus, *Zeb1* acts as a tumor suppressor in *MLL-AF9* and *Meis1a/Hoxa9* AML LSCs.

## Discussion

*Zeb1*, in its capacity as a critical EMT regulator, controls myriad processes in embryonic development and, through the agency of tissue-specific stem cells, acts as a critical regulator of adult tissue homeostasis (18). Deregulation of *Zeb1* activity has been implicated in multiple cancer types and, in these settings, *Zeb1* acts as an instigator of the activity of cancer stem cells, a subset of cancer cells driving therapy resistance and metastasis, which ultimately cause fatality (42, 43). Understanding of the cellular and molecular mechanisms underpinning *Zeb1*-mediated regulation of stem cell self-renewal, lineage fate, and differentiation in normal and cancer stem cells remains incomplete. Here, in stem cells of the hematopoietic system, we find that acute conditional deletion of *Zeb1* causes a profound cell-autonomous self-renewal defect and differentiation block across all lineages after transplantation and deregulates a transcriptional program associated with cell polarity. Strikingly, acute conditional deletion of *Zeb1* in HSCs and their progeny affects the lineage fate and cell survival of T cells, leading to a rapid loss of thymocytes and CD8^+^ T cell subsets.

While it is known that *Zeb1* and other TFs, such as zinc finger TF *Gata3*, are essential in T cell development (67, 68), the process in adults is less clear. Here, we identify *Zeb1* as an indispensable regulator of transcriptional programming for the entire adult T cell repertoire, during initial T cell commitment from HSPC BM progenitor subsets through to cell survival during positive and negative selection in the thymus. In spite of incomplete gene deletion using the *Mx-Cre* system, we also identified *Zeb1* as a regulator of CD8^+^ EM in BM and PB and CD8^+^CM T cells from spleen, supporting previous observations that *Zeb1* is critical to CD8^+^ T cell function during infection (69). Our data are also consistent with the notion that the ZEB family member *Zeb2* plays reciprocal roles in CD8^+^ T cell biology (69) and that it does not compensate for the absence of *Zeb1*. Further understanding *Zeb1*-mediated control of adult T cell differentiation may have implications for immunosurveillance, a naturally occurring immune mechanism involving CD8^+^ T cells and other immune subsets that eradicate tumor cells (70, 71). In particular, the complex interplay between the necessity for *Zeb1* in immune cell subsets involved in immunosurveillance and the tumor microenvironment, where paradoxically, ZEB1 expression can drive metastasis by interfering with immune checkpoints, requires further exploration to negate possible toxic effects associated with targeting ZEB1 or ZEB1 target genes therapeutically in cancer. Nonetheless, the benefits of modulating the *Zeb1* transcriptional/epigenetic network in cancer immunotherapy have been clearly illustrated in the blockade of CD47, a direct transcriptional target of *Zeb1*, that enhances phagocytosis of breast cancer cells undergoing EMT (72).

*Zeb1^–/–^* HSCs were functionally defective in their capacity to generate other blood lineages in transplantation, suggesting that *Zeb1* modulates the ability of HSCs to differentiate correctly in vivo through repression of lineage-commitment–affiliated gene programs in HSCs. In keeping with the notion of lineage-specific transcriptional repression licensed by *Zeb1*, we observed upregulation of 79% of genes in *Zeb1^–/–^* HSCs together with a robust gene expression signature associated with deregulated multilineage differentiation. Relatively few transcriptional repressors, including *Gfi1* and *Gfi1b*, have been shown to regulate HSC self-renewal and differentiation function (73). *Gfi1^–/–^* HSCs have a phenotype resembling that observed in *Zeb1^–/–^* HSCs (74, 75), and notably, both *Gfi1* and *Gfi1b* were transcriptionally repressed in *Zeb1^–/–^* HSCs, suggesting positive regulation by ZEB1. *Zeb1* therefore likely acts as a transcriptional repressor that regulates HSC self-renewal and global differentiation via a transcriptional repressor network that includes both *Gfi1* and *Gfi1b*.

*Zeb1* regulates HSC self-renewal and differentiation in association with a transcriptional program of cell polarity, which relates to the structural and cellular changes that occur to a cell, facilitating specialized function, such as cell division, adhesion, or migration (76). Several studies in *Drosophila melanogaster* male germline stem cells (77, 78) support the longstanding hypothesis that cell polarity acts as a critical mechanism that asserts control of symmetric versus asymmetric stem cell division and therefore stem cell fate, simply put, striking a balance between self-renewal and differentiation fates in tissue homeostasis and under conditions of physiologic stress (76, 79). Notably, *Numb*, a marker of asymmetric division in *Drosophila melanogaster* neuroblasts (80), and *Crb3*, which asymmetrically distributes polarity proteins in mouse preimplantation embryos (81), were deregulated in *Zeb1^–/–^* HSCs. Taken together with the observation that *Zeb1^–/–^* HSCs have decreased self-renewal potential, these data suggest that *Zeb1^–/–^* HSCs favor symmetric, differentiating divisions over asymmetric divisions. Other regulators of cell polarity, including genes associated with apical-basal polarity (82), such as tight junctions (*Ocln*, *Marveld2*, *Tjp3*), adherens junctions (*Cdh1*) and desmosomes (*Krt8*, *Dsc2*, *Dsg2*), were upregulated in *Zeb1^–/–^* HSCs. We provide evidence that derepression of EpCAM in *Zeb1^–/–^* HSCs correlates with enhanced HSC survival in homeostasis and transplantation. Yet enhanced HSC survival and relative upregulation of EpCAM in PB correlate with a block in engraftment in transplantation of *Zeb1^–/–^* HSCs, suggesting that enhanced survival of HSCs mediated by EpCAM disturbs the delicate balance of self-renewal versus differentiation fates.

EpCAM expression in *Zeb1^–/–^* HSCs reduced mitochondrial metabolism, which is also important for HSC self-renewal, cell survival,and differentiation fates (57). For example, reduced pyruvate metabolism was observed in *EpCAM^+^* *Zeb1^–/–^* HSPCs, which is consistent with evidence that ablating aspects of pyruvate metabolism causes HSC exhaustion and a block in HSC differentiation (83, 84). Associated with a low metabolic state, *EpCAM^+^* *Zeb1^–/–^* HSPCs also exhibited reduced ribosome biogenesis, which may reflect enhanced cell survival mediated by the combinatorial lack of p53 target gene phosphorylation, stabilization of p53, and reduced rRNA observed in *EpCAM^+^* *Zeb1^–/–^* HSPCs (58, 85). In future work, it will be of interest to further evaluate the genetic requirement for EpCAM in *Zeb1*-mediated HSC function in the context of mitochondrial metabolism and ribosome biogenesis.

Deregulation of cell adhesion molecules or other cell polarity genes, such as EpCAM, which are a normal feature of epithelial cells, could also be incompatible with the predominantly mesenchymal milieu of the BM environment and may facilitate aberrant, cell-autonomous–driven interactions of HSCs with components of the BM niche (86). This could restrain the motility or alter survival and quiescence of HSCs (or HSPC subsets) within the BM. Studies should be conducted using in vivo imaging of *Zeb1^–/–^* HSCs in their BM habitat to define the broader role of cell polarity–related molecules in vascular and osteoblastic BM niches and how they might influence HSC fate.

Perturbation of cell polarity is also a hallmark of cancer development, (87) and given our findings of *Zeb1’s* impact on myeloid differentiation — which is blocked in AML — we explored the function of *Zeb1* in AML. In hematologic malignancies, *Zeb1* has variably been reported as either a tumor suppressor (in Sézary syndrome and adult T cell leukemia/lymphoma) (88–90) or an oncogene (in mantle cell lymphoma) (91). We found *ZEB1* expression was reduced in select AML patient subtypes, including those involving *MLL* chromosomal translocations that confer poor prognosis in AML (92). Induction of *Zeb1* KO in LSC mouse models of *MLL-AF9*– and *Meis1a/Hoxa9*-driven AML accelerated disease progression, implying that *Zeb1* acts as a tumor suppressor in AML LSCs. In support of this, ablating prooncogenic *Gata2* in AML LSCs caused upregulation of *Zeb1* expression (66). Taken together with analysis of *Zeb1^–/–^* HSCs, these data suggest that *Zeb1*-mediated control of HSC self-renewal, apoptosis, and differentiation fates is integral to suppressing the vulnerability of HSCs to leukemic transformation and disease progression in AML. This view is in agreement with the likely preleukemic selective advantage provided by decreased ribosome biogenesis (58) and enhanced AML signaling in *Zeb1^–/–^* *EpCAM^+^* HSPCs. In contrast, however, and consistent with an oncogenic function for *Zeb1* in AML, high expression of *Zeb1* has been found to drive dissemination of AML LSCs and leukemic cells to extramedullary sites and other organs (92). Yet the cell context–dependent requirement for *Zeb1* in initiating and propagating AML (93) remains ambiguous and requires further in-depth experimentation.

## Methods

### Mice.

We generated *Zeb1^fl/fl^* mice (23) which were bred with *Mx1-Cre*^+/–^ mice (25) to generate an experimental cohort of *Zeb1*^fl/fl^*;Mx1-Cre*^–/–^ (control) and *Zeb1^fl/fl^;Mx1-Cre*^+/–^ (*Zeb1*^–/–^). *Zeb1* was deleted after i.p. administration of pIpC (6 doses every alternate day, 0.3 mg per dose, GE Healthcare). Genotyping is described in Supplemental Methods.

### Flow cytometry analysis.

Bones (femurs, tibias, iliac bones) were crushed using a pestle and mortar in PBS supplemented with 2% FBS, and the BM cell suspension was filtered through a 70 μm cell strainer (Miltenyi Biotec). Spleen and thymi were minced through a 70 μm cell strainer to obtain a homogeneous cell suspension. PB was obtained from the tail vein in EDTA-treated tubes (Starstedt). Red blood cells were lysed by ammonium chloride solution (STEMCELL Technologies). For the immunophenotypic analysis, cells were stained as follows: HSPCs (LSK SLAM): lineage cocktail was prepared from a pool of biotinylated antibodies of differentiated cell markers in PBS 2% FBS (MAC1 and GR1 for myeloid cells, TER119 for erythroid lineage, B220 for B cells, CD3e, CD4, CD8a for T cells), SCA-1-APCCy7, C-KIT-APC, CD150-PECy7, and CD48-FITC to study HSC, MPP, HPC1, and HPC2; for the committed progenitors (LK), LIN cocktail as in LSK SLAM, SCA-1-APCCy7, CKIT-APC, CD34-FITC, CD16/32-PECy7, CD135-PE, and CD127-BV650 to study LMPP, CMP, GMP, MEP, and CLP. The lineage cocktail was detected by adding streptavidin as a secondary antibody. Lineage-positive cells from the BM and spleen were stained for GR1-PECy7 and MAC1-APC (myeloid cells), CD3-APC, CD4-PE, CD8-APCCy7 (T cells), B220-FITC (B cells), CD62L-PECy7, and CD44-APC (naive, effector, and memory T cells). For thymocytes, cells were stained for CD4 and CD8, CD44, CD25, and C-KIT to study early and late stages of T cell development in thymus. For apoptosis assay, after staining the cells for cell-surface markers, they were stained with annexin V–PE antibody (BioLegend) for 30 minutes in the dark at room temperature (RT), and DAPI (1 μg/mL) (Molecular Probes) was added before running the samples. Ki67 for cell-cycle analysis in HSCs and intracellular staining were done after the extracellular staining, cells were fixed in 1% paraformaldehyde (PFA) (Thermo Fisher) for 20 minutes at 4°C, permeabilized using PBS containing 0.1% Saponin (MilliporeSigma) for 30 minutes at 4°C, and then stained with the antibodies for 30 minutes at 4°C in the dark. For cell cycle, cells were incubated with DAPI at a final concentration of 5 μg/mL in the dark for 5 minutes before running the samples. Samples were analyzed using BD LSRFortessa (BD Biosciences). Data were analyzed using FlowJo, version 10.0.8 (Tree Star). A full list of antibodies used is shown in Supplemental Table 1.

For HSC sorting, BM cell suspension was obtained and red blood cells were lysed by ammonium chloride solution (STEMCELL Technologies). Cells were enriched for CKIT by MACS (Miltenyi Biotec) using anti-CKIT magnetic beads (Miltenyi Biotec). CKIT^+^ cells were stained as described earlier, and HSCs were sorted using a BD FACSAria Fusion (BD Biosciences).

### Transplantation experiments.

C57BL/6 SJL mice (CD45.1) were used as recipients for all the transplantations, except that the niche transplantation C57BL/6 (CD45.2) mice (The Jackson Laboratory) were used as recipients. The mice were lethally irradiated at 9 Gy (split dose). For primary transplantation, 150 HSCs from *Zeb1^–/–^* and control cells mixed with 2 × 10^5^ whole BM (CD45.1) (supporting cells) were i.v. transplanted into lethally irradiated mice (CD45.1). To monitor the engraftment, tail-vein bleeding was performed at different time points after transplant. To further assess the capacity of Zeb1-deficient HSCs to repopulate secondary recipients, 300 HSCs from *Zeb1^–/–^* and control mixed with 3 × 10^5^ whole BM (CD45.1) (supporting cells) were i.v. transplanted into lethally irradiated mice (CD45.1). The engraftment ability was monitored via tail-vein bleeding as was done with the primary recipients.

For cell-autonomous transplantation, *Zeb1* was deleted specifically in hematopoietic cells (but not in BM niche cells) after transplanting 5 × 10^5^ whole BM (CD45.2) from *Zeb1^fl/fl^;Mx1-Cre^+^* and *Zeb1^fl/fl^;Mx1-Cre^–^* along with 5 × 10^5^ whole BM (CD45.1) (supporting cells) into lethally irradiated recipients (CD45.1). Six weeks later, 6 doses of pIpC (every alternate day, 0.3 mg per dose) were i.p. injected to delete *Zeb1*. Mice were dissected at day 14 after the last dose of pIpC and analyzed. For cell-autonomous secondary transplantation, 5 × 10^5^ CD45.2 donor BM cells were sorted from control and *Zeb1^–/–^* primary recipients and mixed with competitor cells and retransplanted into lethally irradiated recipients.

For niche transplantation, 1 × 10^6^ total BM cells from WT CD45.1^+^ mice were transplanted into lethally irradiated *Zeb1^fl/fl^;Mx1-Cre^+^* and *Zeb1^fl/fl^;Mx1-Cr^–^* mice. Six weeks later, 6 doses of pIpC (every alternate day, 0.3 mg per dose) were i.p. injected to delete *Zeb1*. Mice were dissected at week 16 after the last dose of pIpC.

### Leukemia transformation assay.

1 × 10^6^ CD45.2^+^C-KIT^+^ cells were obtained from control and *Zeb1^fl/fl^;Mx1-Cre^–/+^* mice and cultured in IMDM 10% FBS supplemented with 40 ng/mL SCF, 20 ng/mL IL-3, and 20 ng/mL IL-6. The next day, the cells were transduced with retroviral vectors encoding Meis1a/Hoxa9 and MLL-AF9 using retronectin-coated plates (TaKaRa) as described previously (64, 66). After 72 hours, 5000 cells were plated in colony-forming units assay 1 (CFC1) using MethoCult M3231 (STEMCELL Technologies) semi-solid media for 6 days, and this process was repeated for up to 3 rounds of CFCs. At the end of CFC3, pre-LSCs were harvested and sorted according to C-KIT expression and transplanted into lethally irradiated primary recipients. Three weeks later, *Zeb1* was deleted after i.p. administration of pIpC (8 doses every alternate day, 0.3 mg per dose, GE Healthcare). Mice were monitored for AML development.

### RNA-Seq.

RNA from HSCs (LSK CD150^+^CD48^–^) and CLPs (LIN^–^ SCA-1^lo^C-KIT^lo^CD127^+^) from control and *Zeb1^–/–^* mice 14 days after the last dose of pIpC injection was extracted using the RNAeasy Micro Kit (QIAGEN). Total RNA quality and quantity were assessed using Agilent 2100 Bioanalyzer and the RNA Nano 6000 Kit (Agilent Technologies). The library was prepared using the NEB Ultra II Directional RNA Library Prep Kit for Illumina. The libraries then were sequenced using a 75 base paired end (2 × 75bp PE) dual index read format on the Illumina HiSeq4000 according to the manufacturer’s instructions. Further details on sequencing and bioinformatics were described previously (66).

The heatmap was created using Morpheus, an online tool, (Broad Institute). DEGs with an FDR of less than 0.05 were used for heatmaps. The biological pathway analysis was performed using BioCarta, KEGG, and Reactome pathway databases run on GSEA software (94) as well as IPA software (QIAGEN). IPA was used to create a prediction network of Zeb1 interactions with its target genes.

RNA-Seq for EPCAM^+^ and EPCAM^–^ from *Zeb1^–/–^* mice is described in Supplemental Methods.

All RNA-Seq data are available in the NCBI’s GEO database (GSE153664, GSE154615).

### AML patients bioinformatic analysis.

ZEB1 expression data were analyzed from a cohort of 2611 AML patients and 77 controls obtained from the GEO database (GSE14468, GSE22845, GSE10358, GSE12417, GSE13159, GSE14062, GSE15434, GSE16015, GSE38987, GSE22056, GSE33223, GSE17855, GSE15389) (95) and ArrayExpress (E-MTAB-3444) (96). R software was used to analyze and produce data. Data processing, normalization, and analysis were described previously (97).

### Statistics.

Figures were prepared using Prism (GraphPad Software). Statistical analyses were done using Mann-Whitney *U* test to calculate significance, which was defined as *P* < 0.05\.

### Study approval.

All animal experiments were performed according to protocols ratified by the UK Home Office and carried out at Cardiff University animal facility under project number 30/3380.

## Author contributions

A Almotiri designed and performed experiments, analyzed and interpreted data, prepared the figures, and contributed to writing the manuscript. HA contributed to experimental design, performed experiments, analyzed and interpreted data, analyzed RNA-Seq data, and reviewed the manuscript. JBMG contributed to experimental design, performed experiments, analyzed and interpreted data, and reviewed the manuscript. A Abdelfattah and BA performed experiments, analyzed data, and reviewed the manuscript. LS, A Greene, MG, A Gibbs, A Alsayari, ST, LAT, and DS performed experiments and contributed to data analysis. SE and PG performed bioinformatics analyses. MPS, SB, and TB contributed to experimental design and analysis and reviewed the manuscript. ASB and FAS contributed significantly to experimental design, data analysis and interpretation, and manuscript preparation. NPR conceived and supervised the project, designed experiments, analyzed and interpreted the data, and wrote the manuscript.

## Supplementary Material

Supplemental data

## Version 1 10/27/2020

In-Press Preview

## Version 2 01/04/2021

Print issue publication
