# Supplementary material for: Zeb1 modulates hematopoietic stem cell fates required for suppressing acute myeloid leukemia
Source: J Clin Invest. 2021 Jan 4;131(1):e129115. doi: 10.1172/JCI129115 (PMC7773410; doi:10.1172/JCI129115)

Supplemental Information for

Zeb1 modulates hematopoietic stem cell fates required for suppressing acute myeloid leukemia

Alhomidi Almotiri, Hamed Alzahrani, Juan Bautista Menendez-Gonzalez, Ali Abdelfattah, Badi Alotaibi, Lubaid Saleh, Adelle Greene, Mia Georgiou, Alex Gibbs, Amani Alsayari, Sarab Taha, Leigh-Anne Thomas, Dhruv Shah, Sarah Edkins, Peter Giles, Marc P. Stemmler, Simone Brabletz, Thomas Brabletz, Ashleigh S. Boyd, Florian Siebzehnrubl and Neil P. Rodrigues

Table 1. Antibodies used for flow cytometry analysis.

	Antigen	Clone	Reactivity	Fluorochrome	Dilution	Manufacturer
**HSPCs and committed progenitors**	Pool of lineage+ markers: CD3, CD4, CD8a, B220, MAC1, GR1, Ter119	Shown below individually	Shown below individually	Biotin	CD3 and CD8:1/40, B220 and GR1:1/20, CD4:1/80, MAC1:1/10, Ter119:1/5	Biolegend, eBiosciences
	SCA-1	D7	mouse	APC-Cy7, PE	1/25	Biolegend
	C-KIT	2B8	mouse	APC, PE	1/100	Biolegend
	CD150	TC15-12F12.2	mouse	PE-Cy7	1/100	Biolegend
	CD48	HM48-1	mouse	FITC	1/50	Biolegend
	CD135 (Flt3)	A2F10	mouse	PE	1/50	Biolegend
	CD34	RAM34	mouse	FITC	1/25	ebiosciences
	CD16/32	93	mouse	PE-Cy7	1/25	Biolegend
	CD127 (IL-7rα)	A7R34	mouse	BV650, PE	1/100	Biolegend
**Erythroid lineage**	Ter119	TER-119	mouse	APC-Cy7	1/1000	Biolegend
**T cell panel**	CD3	17A2	mouse	APC, FITC	1/1000	Biolegend
	CD4	GK1.5	mouse	PE, PerCP	1/1000	Biolegend
	CD8a	53-6.7	mouse	APC-Cy7	1/1000	Biolegend
	CD44	IM7	Mouse/ human	APC	1/1000	Biolegend
	CD25	PC61	mouse	PerCP	1/1000	Biolegend
	CD62L	MEL-14	mouse	PE-Cy7	1/1000	Biolegend
**Myeloid cells**	CD11b (MAC1)	M1/70	Mouse / human	APC	1/1000	Biolegend
	GR1	RB6-8C5	mouse	FITC, PE-Cy7	1/1000	Biolegend
**B cells**	B220/CD45R	RA3-6B2	Mouse / human	FITC, APC	1/1000	Biolegend
**Donor/recipient cell staining**	CD45.1	A20	mouse	BV510, BV650, APC	1/500	Biolegend
	CD45.2	104	mouse	BV510, PE, APC-Cy7	1/500	Biolegend
**Cell adhesion molecules**	EpCAM (CD326)	G8.8	mouse	BV711	1/50	Biolegend
	E-Cadherin (CD324)	DECMA-1	Mouse/ human	PE	1/50	Biolegend
	ITGB4 (CD104)	346-11A	mouse	BV711	1/50	BDbiosciences
**Apoptosis**	BCL-XL	54H6	mouse	PE	1/100	Cell signaling
	Annexin V	NA	All mammalian	APC, PE	1/25	Biolegend
	Ki-67	16A8	mouse	APC, PE	1/25	Biolegend
	Fc Block	93	mouse	NA	1/100	Biolegend
	Streptavidin	NA	Mouse / human	Pacific blue, PerCP	1/100	Biolegend, ebiosciences

Zeb1 genotyping

Genomic DNA was isolated from PB, BM and spleen cells from control and *Zeb1*$^{-/-}$ mice using Isolate II Genomic DNA Kit (Bioline) according to the manufacture instructions. The PCR was performed on T100TM Thermal Cycler (Bio-Rad) at the following conditions: for *Zeb1*: volume 25 µL, 95 ^{o}C for 5 min, 95 ^{o}C for 30 sec, 64 ^{o}C for 45 sec, 72 ^{o}C for 1 min 39x, 72 ^{o}C for 5 min, and 10 ^{o}C for ∞; for *Cre*: volume 25 µL, 95 ^{o}C for 3 min, 94 ^{o}C for 30 sec, 55 ^{o}C for 30 sec, 72 ^{o}C for 1 min 30x, 72 ^{o}C for 5 min, and 10 ^{o}C for ∞. The primers that were used were: *Zeb1fl* forward 5′-CGTGATGGAGCCAGAATCTGACCCC-3′; *Zeb1fl* reverse 5′-GCCCTGTCTTTCTCAGCAGTGTGG-3′; *Zeb1* excised reverse 5′-GCCATCTCACCAGCCCTTACTGTGC-3′; Generic *Cre* forward 5′-TGACCGTACACCAAAATTTG-3′; Generic *Cre* reverse 5′-ATTGGCCCTGTTTCACTATC-3′.

Liquid culture assay

2500 LSKs from *Zeb1*$^{-/-}$ *EpCAM*$^{-}$ and *Zeb1*$^{-/-}$*EpCAM*$^{+}$ were sorted. The cells were grown in IMDM liquid culture containing SCF (100 ng/ml), FLT3 (100 ng/ml), and TPO (50 ng/ml) (PEPROTECH) and incubated at 37C for 5 days.

Homing assay

1X10^{7} unfractionated BM cells from *Zeb1*$^{-/-}$ and control mice were transplanted into lethally irradiated mice (CD45.1). 18 hours later, mice were dissected and the BM cells were stained for CD45.1 and CD45.2. The frequency of CD45.2 was analyzed using flow cytometry in BM, spleen, and thymus.

RNA extraction, cDNA Synthesis, and RT-qPCR

For *Zeb1* expression analysis, hematopoietic cells from the bone marrow of 8-12 week old C57/BL6 mice were sorted. RNA extraction from hematopoietic cells was done using RNeasy Micro Kit (Qiagen) according to the manufacturer's instructions. The concentration of extracted RNA was assessed by NanoDrop 2000 Spectrophotometer (Thermo Scientific). Synthesis of cDNA was performed using the Cloned AMV First-Strand cDNA Synthesis Kit (Invitrogen) by T100™ Thermal Cycler (Bio-RAD). To analyze the expression of *Zeb1*, Taqman® Universal Master Mix II (Applied Biosystems) was used and analysis was performed on QuantStudio® 7 Flex Real-Time PCR System (Applied Biosystems). Gene expression was normalized using the housekeeping gene *Hprt*. Three biological replicates were performed on each cell population for both the reference (*Hprt:* Mm00446968_m1) and target (*Zeb1:* Mm00495564_m1) genes.

RNA Sequencing for EpCAM fractions within *Zeb1$^{-/-}$* LSK

Total RNA was isolated from *EpCAM^{+}* and *EpCAM^{-} Zeb1$^{-/-}$* LSK cells 14 days after the last dose of pIpC (n= 4 mice per genotype) using the RNeasy Mini Kit™ (Qiagen) according to the manufacturer's instructions. Total RNA stored in RNase-free water was amplified using SMART-Seq™ v4 Ultra™ Low Input RNA Kit for Sequencing (Takara Bio USA, Inc., Mountain View, USA) with the double strand cDNA (ds-cDNA) being synthesized. Then, ds-cDNA is purified with AMPure XP beads and quantified with Qubit (Life Technologies™). For library preparation, 1 ng cDNA per sample was used as input to construct 250~300 bp insert cDNA libraries using NEBNext® Ultra™ RNA Library Prep Kit (New England Biolabs, Ipswich, USA) following the manufacturer's recommendations. Libraries were sequenced by Novogene, as 150-bp paired-end reads on an Illumina Novaseq 6000 platform.

The quality of raw reads was first assessed using an in-house script. Clean reads were obtained for future analysis after removing reads with low quality, adapter sequences and those sequences containing multiple 'N' bases. Reads were aligned to the mouse genome

(*Mus_musculus*, GRCm38.p6) with STAR (v2.6.1d). The read numbers mapped to each gene was quantified with FeatureCounts (v1.5.0-p3). Fragments Per Kilobase of transcript per Million mapped reads (FPKM) of each gene was calculated based on gene length and mapped read counts. Differential gene expression analysis was performed using the DESeq2 package (v1.20.0) for samples with bio-replicate. Resulting P values were adjusted using the Benjamini and Hochberg approach to control the false discovery rate (FDR, adjusted P value). Genes with an adjusted $P < 0.05$ were deemed differentially expressed. The heatmap was created using Morpheus, an online tool, (Broad Institute). Differentially expressed genes (DEGs) according to FDR <0.05 were used for heatmaps. The biological pathway analysis was performed using BioCarta, KEGG and Reactome pathway databases run on GSEA software (1) as well as Ingenuity Pathway Analysis software (IPA) (QIAGEN Inc., https://www.qiagenbioinformatics.com/products/ingenuity-pathwayanalysis). IPA was used to create the interaction network of anti- and pro-apoptosis genes in HSPCs. Data can be accessed at the GEO (GSE154615).

Supplemental References

1. Liberzon A, Subramanian A, Pinchback R, Thorvaldsdottir H, Tamayo P, and Mesirov JP. Molecular signatures database (MSigDB) 3.0. *Bioinformatics.* 2011;27(12):1739-40.

Supplemental Figure 1

Supplemental Figure 1. (A) Analysis of total cells from BM (n= 13 control and 12 *Zeb1*$^{-/-}$) and spleen (n= 6 control and *Zeb1*$^{-/-}$) as well as spleen weight (n= 8 control and *Zeb1*$^{-/-}$) from control and *Zeb1*$^{-/-}$ mice 14 days after the last dose of pIpC from 4 independent experiments. (B) The frequency of differentiated cells in BM from control and *Zeb1*$^{-/-}$ mice 14 days after the last dose of pIpC from 4 independent experiments (n= 8-12 per genotype)**.** (C) A representative histogram showing the gating of Annexin V analysis in DP thymus cells and the analysis of the frequency of Annexin V^{+} T cells in thymus from control (n=8) and *Zeb1*$^{-/-}$ (n=8) from 4 independent experiments at D14 after the last pIpC dose. (D) A representative histogram showing the gating of Annexin V analysis in DN3 thymus cells and the analysis of the frequency of Annexin V^{+} in DN1-4 populations in thymus from control (n=7) and *Zeb1*$^{-/-}$ (n=6) from 3 independent experiments at D14 after the last pIpC dose. Error bars show mean ± SEM. Mann-Whitney U test was used to calculate significance as follows: *P < .05, **P < .01, ***P < .001, ****P <0.0001.

Supplemental Figure 1

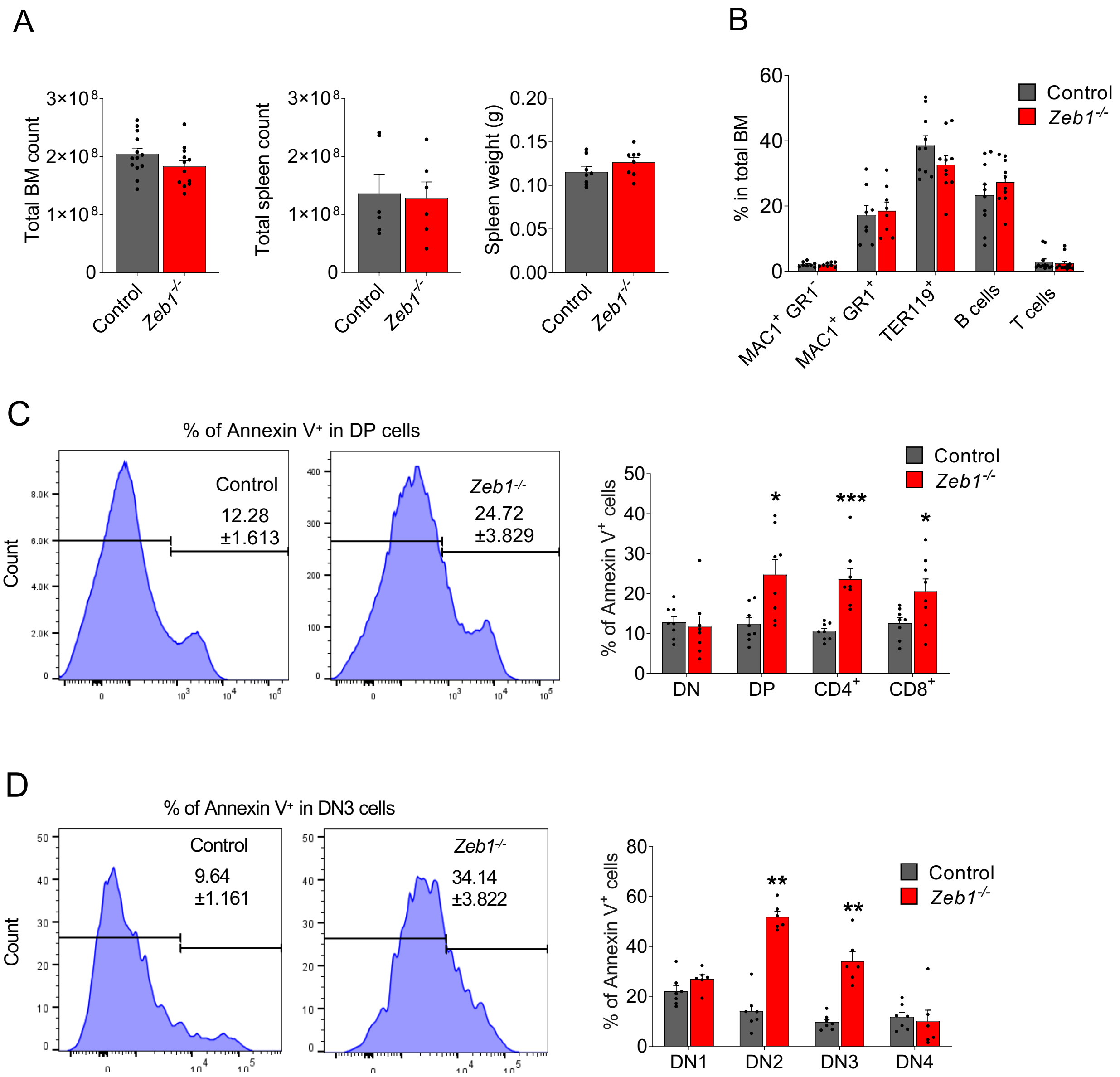

Supplemental Figure 2

Supplemental Figure 2. (A) A scheme of the cell autonomous transplant to assess the effect of *Zeb1* loss in hematopoietic cells but not in niche cells. $5X10^5$ BM cells from *Zeb1*$^{fl/fl}$ *Mx1-Cre*$^{-/-}$ or *Zeb1*$^{fl/fl}$ *Mx1-Cre*$^{+/-}$ (CD45.2) + $5X10^5$ competitor BM cells (CD45.1) were transplanted into lethally irradiated recipients (CD45.1), then six weeks later mice were injected with pIpC to delete *Zeb1* and analysed at 14 days after the last dose of pIpC. The percentage of donor cells in thymus (B) and donor contribution to T cell populations in thymus (C) from control (n=10) and *Zeb1*$^{-/-}$ (n=14) mice from 3 independent experiments via cell autonomous manner. (D) Donor contribution to DN populations (DN1, DN2, DN3, DN4) and ETPs in thymus from control (n= 8 except DN2=7) and *Zeb1*$^{-/-}$ (n=12 except DN2=6) mice from 2 independent experiments via cell autonomous manner. (E) Donor contribution to T cells in peripheral blood (PB), bone marrow (BM), and spleen from control (n=10-11) and *Zeb1*$^{-/-}$ (n=11-12) mice from 2 independent experiments via cell autonomous manner. (F) Representative gel electrophoresis confirming *Zeb1* deletion in BM cells14 days after the last dose of pIpC in the cell autonomous experiment. (G) The frequency of EM T cells within PB CD45.2$^+$ CD3$^+$ CD8$^+$ T cells via cell autonomous manner from control (n= 5) and *Zeb1*$^{-/-}$ (n= 5) mice 14 days after the last dose of pIpC from one experiment. Error bars show mean ± SEM. Mann-Whitney U test was used to calculate significance as follows: *P < .05, **P < .01, ***P < .001, ****P <0.0001.

Supplemental Figure 2

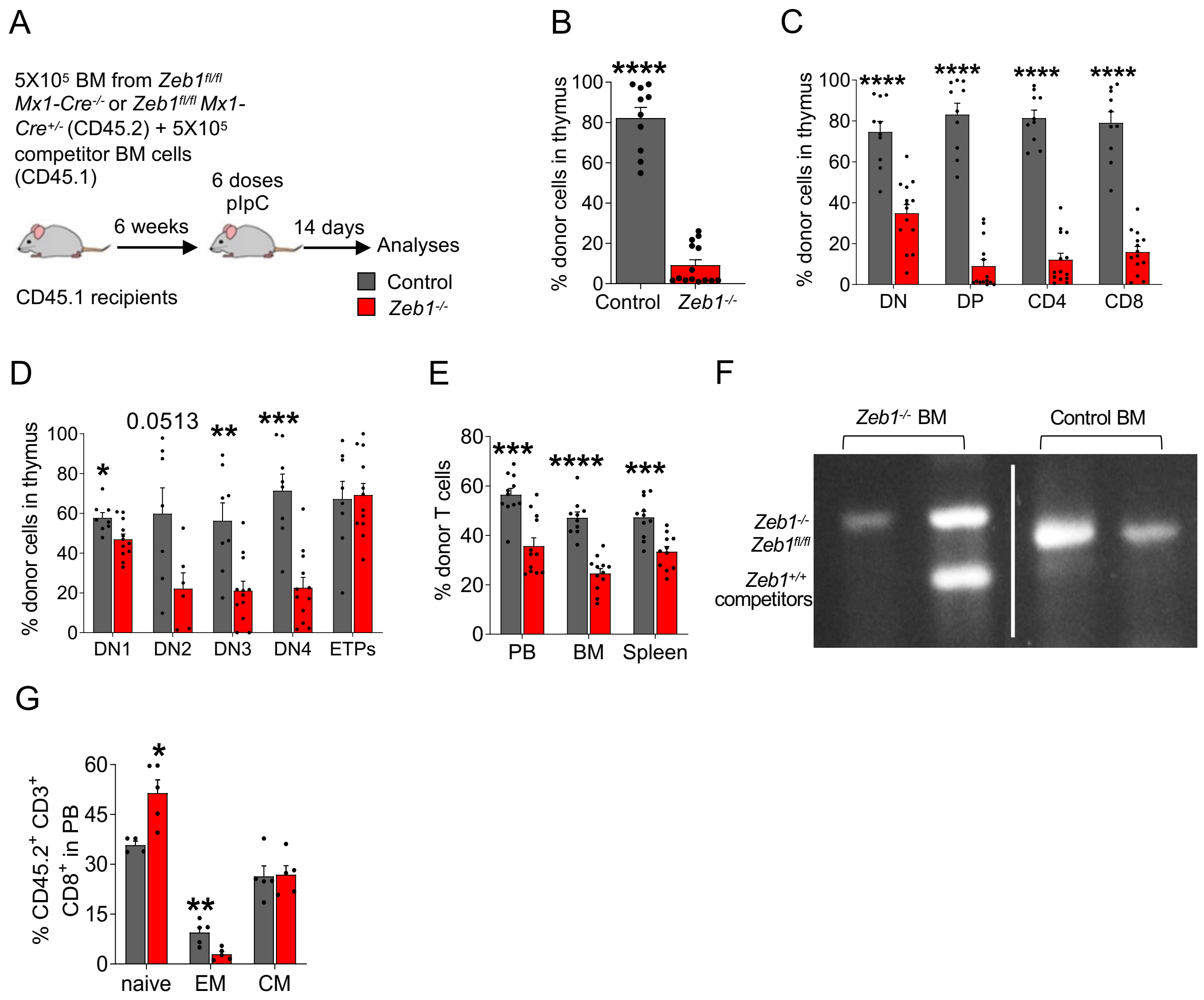

Supplemental Figure 3

Supplemental Figure 3. (A) The frequency of conventional LMPP (LSK $CD34^{+}$ $CD135^{high}$) in BM from control (n=8) and *Zeb1*$^{-/-}$ (n=10) mice 14 days after the last dose of pIpC from 4 independent experiments. The frequency of HSPCs (B) and committed progenitors (C) in BM from control and *Zeb1*$^{-/-}$ mice 14 days after the last dose of pIpC from 3-4 independent experiments (n= 13-14 for HSPCs and n= 6 for committed progenitors). Error bars show mean ± SEM. Mann-Whitney U test was used to calculate significance as follows: *P < .05, **P < .01, ***P < .001, ****P <0.0001.

Supplemental Figure 3

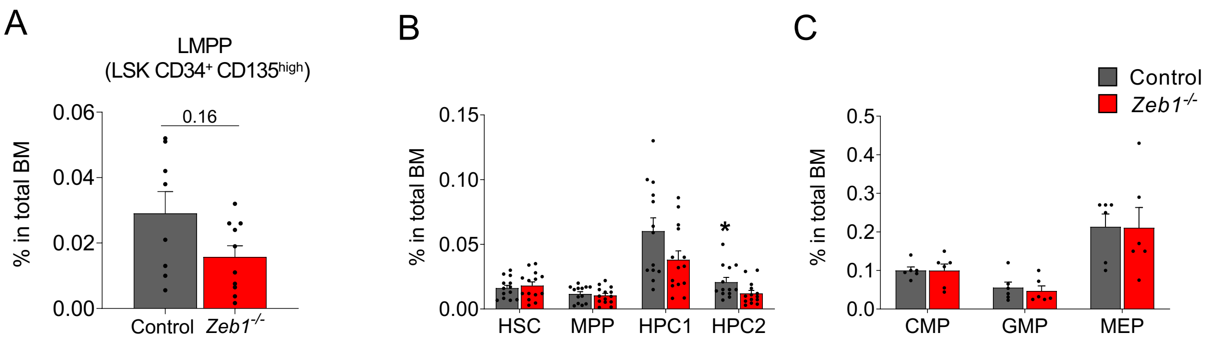

Supplemental Figure 4

Supplemental Figure 4. (A) Schema of homing experiment. $7X10^6$ BM cells from control and *Zeb1*$^{-/-}$ mice were transplanted into lethally irradiated mice then18 hours later, the mice were dissected and homing to the BM cells was checked by flow cytometry. (B) The percentage of donor cells in BM and LSK population 18 hours after BM transplantation from control (n=8 for BM, n=3 for LSK) and *Zeb1*$^{-/-}$ (n=6 for BM, n=2 for LSK) mice. (C) The percentage of donor cells in spleen and thymus 18 hours after BM transplantation from control (n=7-8) and *Zeb1*$^{-/-}$ (n=5). (D) Schema of the experiment to assess the effect of Zeb1-deficient BM niche on hematopoiesis (niche experiment). $1X10^6$ wild type BM cells were transplanted into lethally irradiated *Zeb1*$^{fl/fl}$ *Mx1-Cre*$^{-}$ or *Zeb1*$^{fl/fl}$ *Mx1-Cre*$^{+}$ CD45.2 recipients, then 6 weeks later the mice were injected with pIpC to delete *Zeb1* in BM niche cells and the mice were analysed at week 16. (E) The percentage of PB MAC1^{+} myeloid cells, B220^{+} B cells, and CD4^{+}/CD8^{+} T cells at week 16 post the last pIpC dose of the niche experiment from control (n=10) and *Zeb1*$^{-/-}$ (n= 8) mice from 3 independent experiments. The percentage of HSPCs (F) and committed progenitors (G) in the BM at week 16 post the last pIpC dose of the niche experiment from control (n=6) and *Zeb1*$^{-/-}$ (n=7 for F, n=9 for G) mice from 2 independent experiments. Error bars show mean ± SEM. Mann-Whitney U test was used to calculate significance as follows: *P < .05, **P < .01, ***P < .001, ****P <0.0001.

Supplemental Figure 4

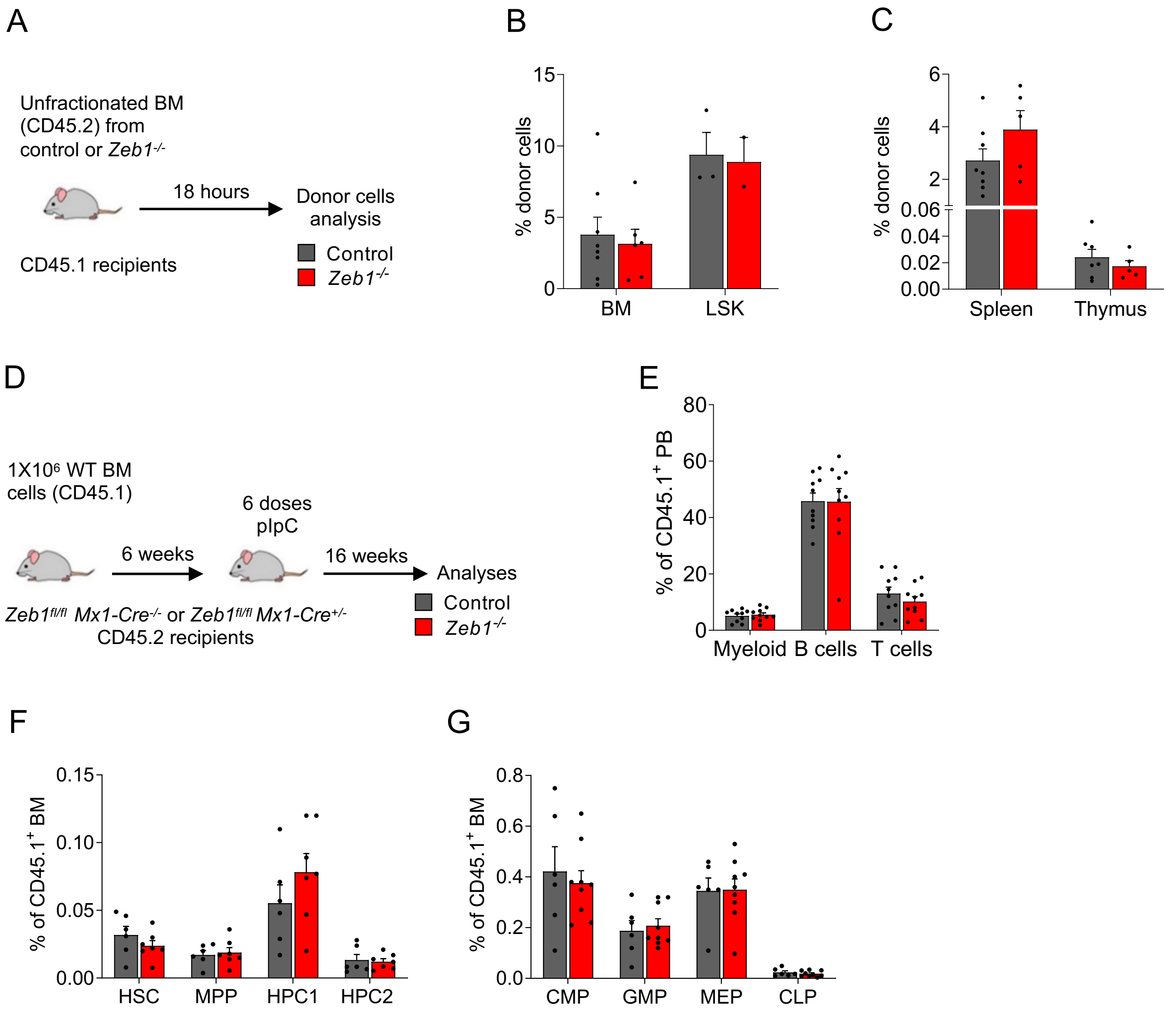

Supplemental Figure 5

Supplemental Figure 5. Representative flow cytometry plots of the expression of E-cadherin (A) and ITGB4 (B) in HSCs (left) and the analysis of their expression in BM HSPCs (right) from control (n=3) and *Zeb1*$^{-/-}$ (n=3) 14 days after pIpC injection. Error bars show mean ± SEM. RNA sequencing was performed in sorted control and *Zeb1*$^{-/-}$ CLPs (Lin^{-} SCA-1^{low} C-KIT^{low} CD127^{+}) 14 days after the last pIpC dose (n=4 for each genotype). (C) Canonical pathways that mostly enriched in *Zeb1*$^{-/-}$ CLPs derived from the BioCarta, KEGG and Reactome pathway databases. Data is shown as –log10 (p-value), and the dashed black line indicates p-value = 0.05, the analysis was performed using the GSEA software. (D) GSEA plots representing tight junction organization and cell-cell Junction assembly phenotype enriched in *Zeb1*$^{-/-}$ CLPs. (E) Heat maps represent the differentially expressed genes within control and *Zeb1*$^{-/-}$ CLPs related to T cell, cytoskeleton and cell adhesion. The heatmap scale represents Z- score. (F) Analysis of the overlap between the DEGs from HSC RNA-Seq and CLP RNA-Seq using IPA software. A total of 47 genes were differentially expressed in *Zeb1*$^{-/-}$ CLPs and 222 genes were differentially expressed in *Zeb1*$^{-/-}$ HSCs. 2 DEGs from *Zeb1*$^{-/-}$ HSCs were excluded by the IPA software.

Supplemental Figure 5

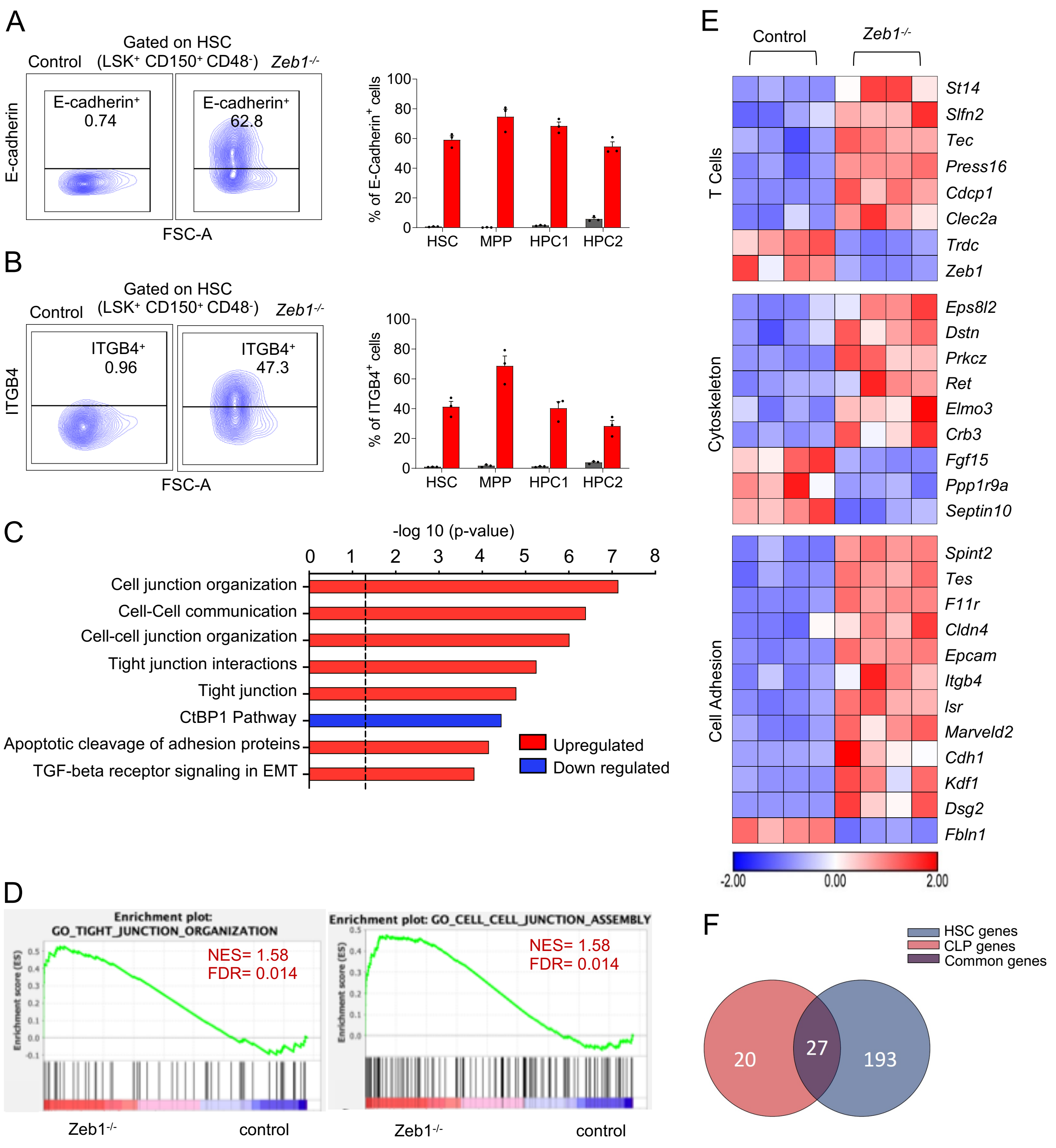

Supplemental Figure 6

Supplemental Figure 6. (A) An IPA interaction network of EpCAM and some apoptotic genes that were differentially expressed in *Zeb1*$^{-/-}$ *EpCAM*$^{+}$ vs *Zeb1*$^{-/-}$ *EpCAM*$^{-}$ and their effect on apoptosis process in HSCPs. Functions colored with blue means inhibition and those with red means activation.

Supplemental Figure 6

A

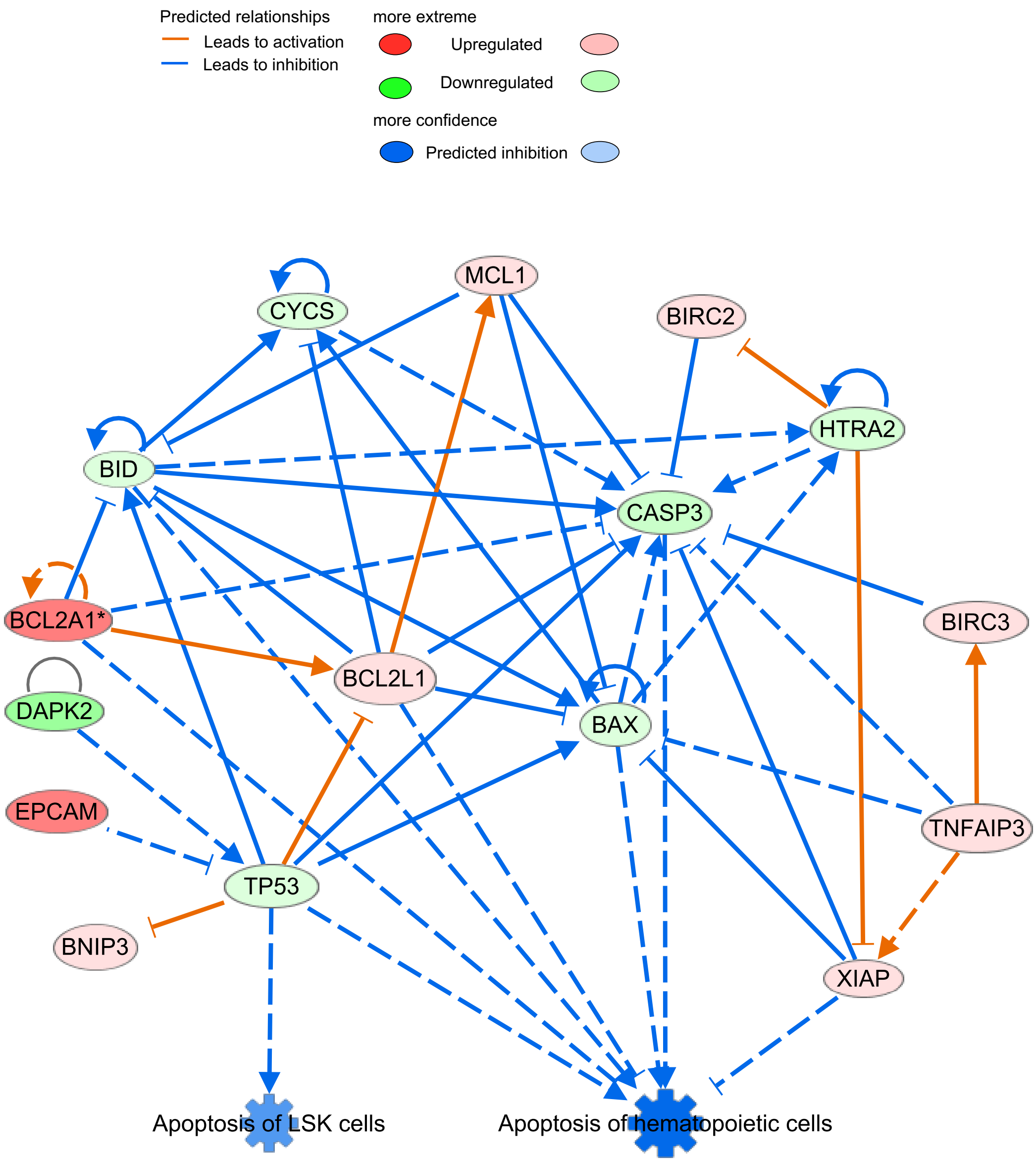

Supplement: Supplemental data [file jci-131-129115-s507.pdf]
